# The virtue of training: extending phage host spectra against vancomycin-resistant *Enterococcus faecium* strains using the Appelmans method

**DOI:** 10.1128/aac.01439-23

**Published:** 2024-04-09

**Authors:** Julien Lossouarn, Elsa Beurrier, Astrid Bouteau, Elisabeth Moncaut, Maria Sir Silmane, Heïdi Portalier, Asma Zouari, Vincent Cattoir, Pascale Serror, Marie-Agnès Petit

**Affiliations:** 1 Université Paris-Saclay, INRAE, AgroParisTech, Micalis Institute, Jouy-en-Josas, France; 2 CHU de Rennes, Service de Bactériologie-Hygiène Hospitalière et CNR de la Résistance aux Antibiotiques (laboratoire associé "Entérocoques"), Rennes, France; 3 Université de Rennes, INSERM, UMR_S1230 BRM, Rennes, France; Johns Hopkins University School of Medicine, Baltimore, Maryland, USA

**Keywords:** virulent phages, host range extension, Appelmans protocol, *Enterococcus faecium*, vancomycin-resistant enterococci

## Abstract

Phage therapy has (re)emerged as a serious possibility for combating multidrug-resistant bacterial infections, including those caused by vancomycin-resistant *Enterococcus faecium* strains. These opportunistic pathogens belong to a specific clonal complex 17, against which relatively few phages have been screened. We isolated a collection of 21 virulent phages growing on these vancomycin-resistant isolates. Each of these phages harbored a typical narrow plaquing host range, lysing at most 5 strains and covering together 10 strains of our panel of 14 clinical isolates. To enlarge the host spectrum of our phages, the Appelmans protocol was used. We mixed four out of our most complementary phages in a cocktail that we iteratively grew on eight naive strains from our panel, of which six were initially refractory to at least three of the combined phages. Fifteen successive passages permitted to significantly improve the lytic activity of the cocktail, from which phages with extended host ranges within the *E. faecium* species could be isolated. A single evolved phage able to kill up to 10 of the 14 initial *E. faecium* strains was obtained, and it barely infected nearby species. All evolved phages had acquired point mutations or a recombination event in the tail fiber genetic region, suggesting these genes might have driven phage evolution by contributing to their extended host spectra.

## INTRODUCTION

Enterococci are ubiquitous gram-positive facultative anaerobic bacteria commonly found as commensal of the mammalian intestine (1, 2). Within the human gastrointestinal tract, the most prevalent enterococcal species are *Enterococcus faecalis* and *Enterococcus faecium*, representing up to 1% of the human gut microbiota (3, 4). However, these two species may colonize up to high densities and behave as opportunistic pathogens in immunocompromised patients with antibiotic-induced microbiota dysbiosis. *E. faecalis* and *E. faecium* have thus emerged as a leading cause of hospital-acquired infections and are mostly responsible for urinary tract infections, peritonitis, bacteremia, or endocarditis (5, 6).

The serious threat posed by enterococci is mainly related to their antibiotic resistance. The first wave of hospital-associated enterococci infections, mainly caused by *E. faecalis*, appeared in the USA in the late 1970s and mirrored the introduction of the third-generation cephalosporins to which all enterococci are intrinsically resistant (6, 7). The second wave of infections which we are now facing began in US hospitals in the early 1990s and spread worldwide with the increasing use of vancomycin and broad-spectrum antibiotics (6, 7). In this context, *E. faecium* has progressively become the most therapeutically challenging enterococcal species, being able to acquire and express antimicrobial resistance genes more frequently than *E. faecalis* (6, 7). This led to the adaptation of specific clinical *E. faecium* clones resistant to high levels of β-lactams and vancomycin, forming a single clonal complex, CC17, within which the sequence types 17, 78, and 18 (ST17, ST78, and ST18) are widespread genetic lineages (7, 8). This prevalent and rapid spread of vancomycin resistance in *E. faecium* is of particular concern. It has led the World Health Organization to classify the species as a “high priority” in its list of multi-drug resistant (MDR) bacterial pathogens, against which new drugs are urgently needed (9). Given the inevitable emergence of bacterial resistance to new antimicrobial molecules, alternative therapeutics are also an important avenue of research, to combat vancomycin-resistant enterococci (VRE), and *E. faecium* (VREfm) in particular.

Although neglected for a long time, except within the former Soviet Union area, virulent bacteriophages (phages), i.e., viruses that infect and obligatorily lyse bacteria, are nowadays considered worldwide to combat MDR bacteria (9–11). Initial studies on enterococci were directed against *E. faecalis* and more recently turned toward *E. faecium,* involving either phage particles (12–17) or phage endolysins (18, 19), as well as the combinations of phage and antibiotics (20–24). One successful case of phage therapy was recently reported in an infant suffering from a VREfm intra-abdominal infection (25). This encouraging result underlines the potential usefulness of phage therapy and the need to pursue the isolation and characterization of virulent phages targeting the CC17. It is, however, not always possible to isolate phages that target the most relevant clinical isolates. In order to cope with this constraint, one of the approaches, known as the Appelmans technique, has been designed and successfully used for a long time in Georgia at the Eliava Institute of Bacteriophage, Microbiology and Virology (IBMV). This technique was recently brought back into the spotlight in Western journals and consists of successive passages of a phage cocktail on a set of naive strains that are initially mostly phage refractory and exposed individually to the cocktail until they lyse. This method is designed to (re)boost therapeutic phage cocktails by generating phages with broader host spectra than the initial phage isolates (26, 27).

In the present work, we first isolated a collection of 21 virulent phages against *E. faecium*, selected for their capacity to multiply in CC17 isolates, and determined their plaquing host range. After completely characterizing the five phages displaying the best and complementary lytic activities, we focused on broadening their host range using the Appelmans strategy. Four of the phages were thus combined in cocktail and evolved by iteratively growing them on a set of eight distinct and previously unexposed VREfm strains that were mostly resistant to each phage. Following 15 passages of the cocktail, the resulting host range and the mutations of the evolved phages were investigated.

## MATERIALS AND METHODS

### Sample for bacteriophage isolation, bacterial strains, and growth conditions

Four raw sewage water samples from Ile-de-France region (France), as well as 12 pig fecal virome samples (28), were used as sources of phage. A total of 14 *E. faecium* clinical isolates (detailed in Table S1) from the National Reference Center for enterococci (Rennes, France) were used for phage isolation. All these strains were statically cultivated aerobically in brain-heart infusion (BHI) liquid medium at 37°C.

### Whole-genome sequencing of the 14 VREfm isolates, phylogenetic context, multilocus sequence typing, and resistome analyses

Bacterial genomic DNA was isolated with the Quick-DNA Fungal/Bacterial Miniprep Kit (Zymo Research, Irvine, CA, USA). DNA libraries were prepared with the NEBNext Ultra DNA Library Prep Kit for Illumina (New England Biolabs, Ipswich, MA, USA) and sequenced as paired-end reads (2 × 300 bp) on an Illumina MiSeq platform with the MiSeq Reagent Kit version 3. The Illumina reads were *de novo* assembled into a draft genome with SPAdes v3.14.0 software (29), while contigs below 500 bp were discarded. QC parameters used as guidelines were as follows: read depth >60× , N50 >30,000 bp, and number of contigs <300. Then, a phylogenetic analysis based on single-nucleotide polymorphisms (SNPs) was performed with Snippy (https://github.com/tseemann/snippy) using the reference core genome multilocus sequence typing (MLST) of *E. faecium* (1,423 genes) (30) available at “cgmlst.org” website (https://www.cgmlst.org/ncs/schema/991893/). The resulting filtered SNPs were transformed into a distance matrix for tree construction with the neighbor-joining algorithm using Python implementation in “Scikit-bio” package [Tree representations (skbio.tree) — scikit-bio 0.5.9 documentation] and the phylogenetic tree was generated with iTOL v5 (https://itol.embl.de/) (31). Raw reads (in FastQ format) were also submitted to the (Center for Genomic Epidemiology) for MLST and resistome analyses. MLST was performed using the MLST 2.0 server, while identification of acquired resistance genes and chromosomal resistance mutations was carried out using ResFinder v4.1 (with default parameters: 90% identity threshold and 60% minimum length) and LRE-Finder v1.0 (with default parameter: 80% identity threshold) services (32, 33).

### Bacteriophage propagation

The standard double overlay plaque assay technique was performed as described by Jurczak-Kurek and her colleagues (34) with slight modifications. Petri dishes were filled with 25 mL of BHIMgCa medium (BHI supplemented with 10 mM MgSO_4_ and 1 mM CaCl_2_) containing 1.5% agar. One hundred microliters of phage lysates were added to 100 µL of an overnight culture of the targeted strains, and the mix was added to 5 mL of BHIMgCa medium containing 0.3% agarose that was poured onto the surface agar of the Petri dishes. The double agar/agarose plates were incubated for 8 to 24 h at 37°C until plaques appeared.

### Bacteriophage isolation

The one-host enrichment method was used as previously described (34) with minor modifications. Each sewage sample was first centrifuged 10 min at 5,500 g and 4°C. Clarified supernatants were passed through 0.8/0.2 µm pore membrane filters (PALL Corporation Acrodisc PF syringe filter Prod. No. 4187). One milliliter of each filtered sewage sample was then mixed with 1 mL of BHIMgCa 2× and 100 µL of an overnight culture of each one of the 14 bacterial selected strains. Each pig fecal virome sample was similarly used for the enrichment step but only on strain VE14980 (Table S1). All enrichment cultures were incubated 24 h at 37°C and centrifuged 10 min at 8,000 g and 4°C. One hundred microliters of each supernatant (phage lysate) were added to 100 µL of an overnight culture of the strain used in the previous step and plated using the double overlay plaque assay technique. Plaque appearance was screened after 24 h of incubation at 37°C. Each distinct plaque was picked and streaked on an agar base before applying the second layer of BHIMgCa top agarose mixed with the overnight culture of the sensitive strain. The plates were incubated 24 h at 37°C, and each corresponding plaque was picked and streaked again twice to ensure phage purity.

### Bacteriophage concentration

Each resulting plaque was scraped and resuspended in 1 mL of SM buffer (50 mM Tris-HCl pH 7.5, 10 mM MgSO_4_, 1 mM CaCl_2_, 150 mM NaCl, 0.02% gelatin), and the phage suspension was centrifuged 10 min at 8000 g and room temperature. Supernatant was filtered through 0.45 µm (PALL Corporation Acrodisc PF syringe filter Prod. No. 4614) and used to obtain high-titer phage stock [~10^9^ plaque-forming units (PFUs)·mL^−1^] as described by Fortier and Moineau (35). Briefly, 10^4^ to 10^6^ PFUs (depending on phages) were plated using the double overlay method to obtain confluent lysis after 8 h at 37°C. The plates were then flooded with 5 mL of SM buffer and incubated overnight at 4°C. The buffer, in which most phages have diffused, was then carefully recovered, and the phage-containing supernatant was filtered through a 0.45-µm filter.

### Bacteriophage host range

Ten microliters of 100-fold serially diluted high-titer phage stocks were spotted on top of agarose overlays containing overnight culture of different enterococcal species, including *E. faecium*, *E. faecalis*, *Enterococcus durans*, *Enterococcus hirae,* and *Enterococcus mundtii* (Tables S1 and S2)*,* as described above. Plates were incubated at 37°C and examined for plaque appearance 8 and 24 h after spotting. Clear and bull’s eye plaques, obtained from the same dilution rate both on isolation and tested strains (approximately same efficiency of plating), were considered to reflect a strong lytic activity. Clear and bull’s eye plaques obtained at 100-fold lower dilution rate on the test strain compared to the isolation strain were deemed representative of a weak lytic activity as well as whether turbid plaques were obtained regardless of the dilution factor, while no visible plaques indicated an absence of productive lytic activity.

### Bacteriophage examination by transmission electron microscopy

One milliliter of high-titer stocks of phages 2, 4 (Porthos), 7, 16 (Athos), and 23 was ultracentrifuged 2 h at 100,000 g and 4°C (Beckman XL-90; SW 41 Ti rotor). Phage pellets were resuspended in 100 µL of SM buffer. Ten microliters of each phage suspension were spotted onto a Formwar carbon-coated copper grid. Phages were adsorbed to the carbon layer for 5 min, and excess of liquid was removed. Ten microliters of a staining uranyl acetate solution (1%) were then spotted to the grid for 15 s, and excess of liquid was removed again. The grid was imaged at 80 kV in a Hitachi HT7700 transmission electron microscope. The remaining phages were prepared following Ackermann’s procedure (36) with minor modifications. One milliliter of high-titer stocks of phages 6 (dArtagnan), 11, 12, 13, 18 (Aramis), and 27 (Planchet) was centrifuged 1 h at 20,000 g and 4°C. Phage pellets were washed and centrifuged 1 h at 20,000 g and 4°C twice in ammonium acetate buffer (0.1 M, pH 7) before being resuspended in 50 µL of ammonium acetate buffer and examined on grid in transmission electron microscopy as previously described. Measurements of the phage particles were performed on five virions for each phage using ImageJ (37), and average values were given with standard deviations.

### Determination of phage burst size

The one-step growth curves of phages Porthos, dArtagnan, Aramis, Planchet, and Athos were measured using a standard method (38). One milliliter of a log-phase culture of respective isolation strains was centrifuged 5 min at 10,000 g and room temperature and resuspended in 100 µL of prewarmed BHIMgCa. Phage from the high-titer phage stock was added at an MOI of ~0.001 and allowed to adsorb for 5 min at 37°C. The phage/bacteria mix was centrifuged 2 min at 10,000 g and room temperature. The supernatant was titrated to count unadsorbed phage particles, whereas the bacterial pellet was washed in 100 µL of prewarmed BHIMgCa and recentrifuged. The pellet was then suspended and diluted in 10 mL of prewarmed BHIMgCa and cultured at 37°C. Samples were taken at regular intervals and plated at the correct dilution for phage titration. A second set of samples from a synchronized 100-fold diluted culture was taken at same intervals and titrated. For each kinetic, the values indicated the means and standard deviations of three independent experiments.

### Bacteriophage DNA extraction, whole-genome sequencing, functional annotation, taxonomic assignment, and mutation screening

Prior to DNA extraction, 10 mL of high-titer stocks of phages Athos, Aramis, Porthos, dArtagnan, and Planchet as well as the five evolved phages were treated with 40 µL of a nuclease mix (50% glycerol, 0.25 mg·mL^−1^ RNase A, 0.25 mg·mL^−1^ DNase I, 150 mM NaCl) for 30 min at 37°C. Particles were then precipitated by adding 10% (wt/vol) PEG 8000 and 0.5 M NaCl, and let sit overnight at 4°C once PEG was solubilized. Phages were centrifuged 1 h at 5,000 g and 4°C, and resuspended into 500 µL of SM buffer. Insoluble particles were removed by centrifugation (20 s at 12,000 g), and the clarified supernatants were transferred in 1.5 mL microtubes for DNA extraction. One volume of phenol-chloroform-isoamyl alcohol (25:24:1; saturated with 10 mM Tris pH 8.0 and 1 mM EDTA) was added to each sample, and, after gentle mixing for 1 min by inversion, the tube was centrifuged at 10,000 g for 10 min at 4°C. The aqueous phase was recovered, and the phenol-chloroform-isoamyl alcohol extraction repeated. The aqueous phase was then recovered and mixed with one volume of chloroform-isoamyl alcohol (24:1) by gentle inversion and centrifugation as above. The aqueous phase was recovered in 1.5 mL microtube, and DNA was precipitated by adding two volumes of absolute ethanol and 50 µL of sodium acetate (3 M, pH 5.2). Phage DNA was recovered by centrifugation at 13,000 g for 30 min at 4°C. DNA pellet was subsequently washed with 800 µL of 70% (vol/vol) ethanol. After centrifugation at 13,000 g for 10 min at 4°C, the DNA pellet was dried at room temperature for 30 min and dissolved in 50 µL of 10 mM Tris-HCl pH 8.0.

Athos and Aramis DNAs were sequenced with an Ion Torrent platform, whereas Porthos, dArtagnan, and Planchet as well as the five evolved phages were sequenced on the Illumina platform at Eurofins Genomics (HiSeq2500, 5 million of 2 × 150 bp paired-end reads).

To assemble phage genomes, raw reads were quality filtered using Trimmomatic v0.36 (39) dropping reads below 125 b long, cutting bases off the start and/or the end of read if below a threshold quality of 3 and clipping reads with average quality values below 20 on a sliding window of 4 nt, by using the additional option ILLUMINACLIP:TruSeq3-PE.fa:2:30:10 to remove remnant of Illumina adapters and other Illumina-specific sequences from Porthos, dArtagnan, Planchet, and the five evolved phage reads. A total of 50,000 cleaned reads of Athos, Aramis, Porthos, dArtagnan, and Planchet, as well as the five evolved phages, were randomly subsampled with seqtk v1.3 (https://github.com/lh3/seqtk) to facilitate assembly with SPAdes v3.13.0 (29) with -only--assembler and the increasing *kmer* values 21, 33, 55, 77, 99, 127 as parameters. Ten phage contigs were thus obtained, and a 127-bp artifactual direct terminal repeat was removed from Porthos, dArtagnan, Planchet, and the five evolved phage contigs. PhageTerm (40) was used to predict the genomic termini and phage-packaging strategy, using both trimmed Illumina reads and the corresponding assembled contigs. Athos, Aramis, Porthos, dArtagnan, and Planchet genomes were annotated with RAST (41) using genetic code 11, virus option, and RASTtk pipeline. This was followed by a manual annotation in which homologs of the predicted proteins were first searched against the Conserved Domain Database with an *E*-value cutoff of 10^−5^ (42). Remote homology searches were then performed with the Virfam database (43) and using HHblits against the PHROGs database (44) as well as HHPred against the PDB database (45) by only retaining biological function predictions with a probability ≥98% in both cases. All genomic figures were generated using GenoplotR (46). All phage and plasmid genomes used in this study are listed in Table S3. VIRIDIC (47) was used to calculate phage overall intergenomic identities using the recommended thresholds of 95% and ~70% for phages demarcation into species and genera, respectively (48). Putative higher taxonomic affiliations (subfamily/family) were evaluated using VIPTree (49) and GRAViTy (50, 51). The reads from the evolved phages were mapped against the genomes of their corresponding parental phages to search for point mutations, using breseq consensus mode (52). Additional recombination events were searched by global alignments between the evolved and parental phage genomes.

### Isolation and characterization of bacterial phage-resistant mutants

To isolate phage-resistant mutants, 1 mL of high-titer phage stocks of Athos, Porthos, and Aramis was added to 100 µL of overnight cultures of their respective isolation strains (MOI ~50), and these mixes were plated using the double layer technique. Following 20 h of incubation, four colonies arising on each plate were selected and purified by streaking (three times). The phage resistance of the purified colonies was then checked by spot assays, first with the phages used for selection and then with the two other phages, as well as Planchet. Athos, Porthos, and Aramis adsorption rates on their resistant mutants were evaluated as previously described (53). The genomes of the eight phages-resistant mutants corresponding to adsorption mutants (Aramis- and Atos-resistant mutants) were extracted by pelleting, washing, and lysing bacterial cells as previously described (54) and then according to the protocol of the DNeasy Blood & Tissue Kit (Qiagen). To identify genetic mutations linked with the adsorption-default phenotype, point mutations were searched on resistant mutants using the draft genome of the wild-type strain for the mapping (same methods as above for phage genomes). *In vitro* antimicrobial susceptibility testing (AST) was performed on the four Athos-resistant and the four Aramis-resistant mutants as well as one Porthos-resistant mutant. This was done according to the CA-SFM/EUCAST guidelines (www.sfm-microbiologie.org) by the disk diffusion method (ampicillin, imipenem, gentamicin, chloramphenicol, erythromycin, clindamycin, dalfopristin-quinupristin, rifampicin, tetracycline, norfloxacin, and cotrimoxazole) or by minimum inhibitory concentration (MIC) determination by the broth microdilution reference method (Sensititre; Thermo Fisher Scientific, Courtaboeuf, France) for vancomycin, teicoplanin, daptomycin, tigecycline, linezolid, tedizolid, telavancin, dalbavancin, and oritavancin. The frequency of emergence of VE14983 spontaneous colony-forming units (CFUs) was estimated following the plating of 100 µL of an overnight culture of the strain in double overlays in presence of teicoplanin at the MIC of 64 µg/mL, Aramis phage at MOI ~50, or both antibiotic and phage in same respective concentrations simultaneously. After 20 h of incubation at 37°C, CFUs were counted in each case and divided by the number of bacteria initially plated.

### Directed evolution of phages

To extend phage cocktail host spectra, we used the protocol described by Burrowes and his colleagues (27), from the one used in the IBMV from Tbilisi, Georgia and known as “the Appelmans protocol.” We aimed to investigate host range expansion from a phage cocktail, by combining phages Porthos, Aramis, dArtagnan, and Planchet in equal ratio to reach 10^8^ PFUs·mL^−1^. They were iteratively exposed to eight distinct “development” strains mostly initially refractory to individual phage. One hundred microliters of serial 10-fold dilutions (10^0^ to 10^−6^) of the phage cocktail were, respectively, added in each well of seven columns of a 96-well plate and mixed with 100 µL of 100-fold dilution of one distinct bacterial overnight culture per line. Both phages and bacteria dilutions were made in BHIMgCa. For each strain, a well was used as a phage-free growth, while the sterility of the phage cocktail and medium was monitored in two additional wells. The plate was incubated overnight at 37°C and then visually analyzed. The contents of the wells where total and partial lysis occurred, as well as the first one with bacterial growth per line, were recovered, pooled together, then centrifuged 15 min at 5,000 g at 4°C, and filtered through 0.45 µm. This pool of phages, called *n* + 1, was used to perform the next passage in 96-well plate by using the same bacterial strains. We performed 15 such passages.

### Isolation and host range evaluation of evolved bacteriophages

After 15 rounds of phage training, the last (*n* + 15) pooled lysate was checked for novel phages by searching for plaques on the eight development strains. After 24 h growth at 37°C, three plaques per bacterial lawn (with distinct clear plaque morphologies when possible) were selected and purified. High-titer phage stocks of each putative phage variant were then prepared. The phage host spectrum of the evolved phage was established in two steps. First, host ranges were evaluated on the 8 development strains used during the Appelmans experiments, and second, tests were extended to the 6 remaining VREfm strains from the panel (Table S1) as well as 21 enterococci strains from different species (Table S2).

## RESULTS

### Isolation of a bacteriophage collection targeting VREfm isolates

Four wastewater samples were screened for phages forming plaques on 14 VREfm clinical isolates, mostly belonging to the CC17 (Table S1). Twenty phages were obtained, growing on 6 of the initial 14 strains, which all together covered 4 STs (Table S4). Twelve made clear plaques, while five formed turbid plaques, and the three remaining phages made bull’s eye plaques, with greater turbidity toward plaque periphery (34). Phage plaque diameters were ranging from <~1 to ~3 mm (Table S4). In a second round of screening, using pig fecal virome samples, a last phage was isolated, making ~1 mm clear plaques on an additional VREfm isolate belonging to a different ST (Table S4).

To assess phage infectivity, 100-fold serial dilutions of the 21 phage stocks were spotted against the panel of 14 *E. faecium* strains used for the isolation step (Table S1). Three categories of susceptibility profiles were defined. Strong lytic activity corresponded to a phage/strain couple making plaques clear or with a bull’s eye shape with a phage efficiency of plating similar to the reference. Weak lytic activity corresponded to a phage/strain couple forming turbid plaques or clear/bull’s eye plaques but at 100-fold lower dilution rate than the reference. No productive lytic activity when any plaque was visible, and the strain was considered as resistant (Table S5). This revealed 17 distinct phage profiles, with host spectra ranging from 1 to 5 strains and covering together a total of 10 of the 14 VREfm strains and 6 distinct STs (Table S5).

To complete the characterization, 11 out of the 21 phages from the collection were negatively stained and observed by transmission electron microscopy (Fig. S1). The phages 16, 4, 2, 18, 6, 11, 13, 7, 23, 12, and 27 were chosen so as to encompass the different host spectra, prioritizing phages with broader host ranges and strong lytic activities (Table S5). All selected phages belonged to the class of *Caudoviricetes* and corresponded to seven siphophages, two myophages, and two podophages (Fig. S1). Siphophages 6, 12, 18, 11, and 13 have non-contractile tails from ~210 to ~250 nm in length and polyhedral heads with diameters ~ 50 to 60 nm, while siphophages 27 and 23 were larger with a flexible tail ~175 and 450 nm in length and a polyhedral head ~70 and 90 nm in diameter, respectively (Table S6). Myophages 2 and 4 harbored contractile tails ~230 nm in length and polyhedral heads ~100 nm in diameters (Table S6). Finally, podophages 16 and 7 were similar, with short non-contractile tails ~30 nm and slightly elongated polyhedral heads ~40 nm in diameters (Table S6).

### Complete characterization of five selected phages

**Fig 1:**
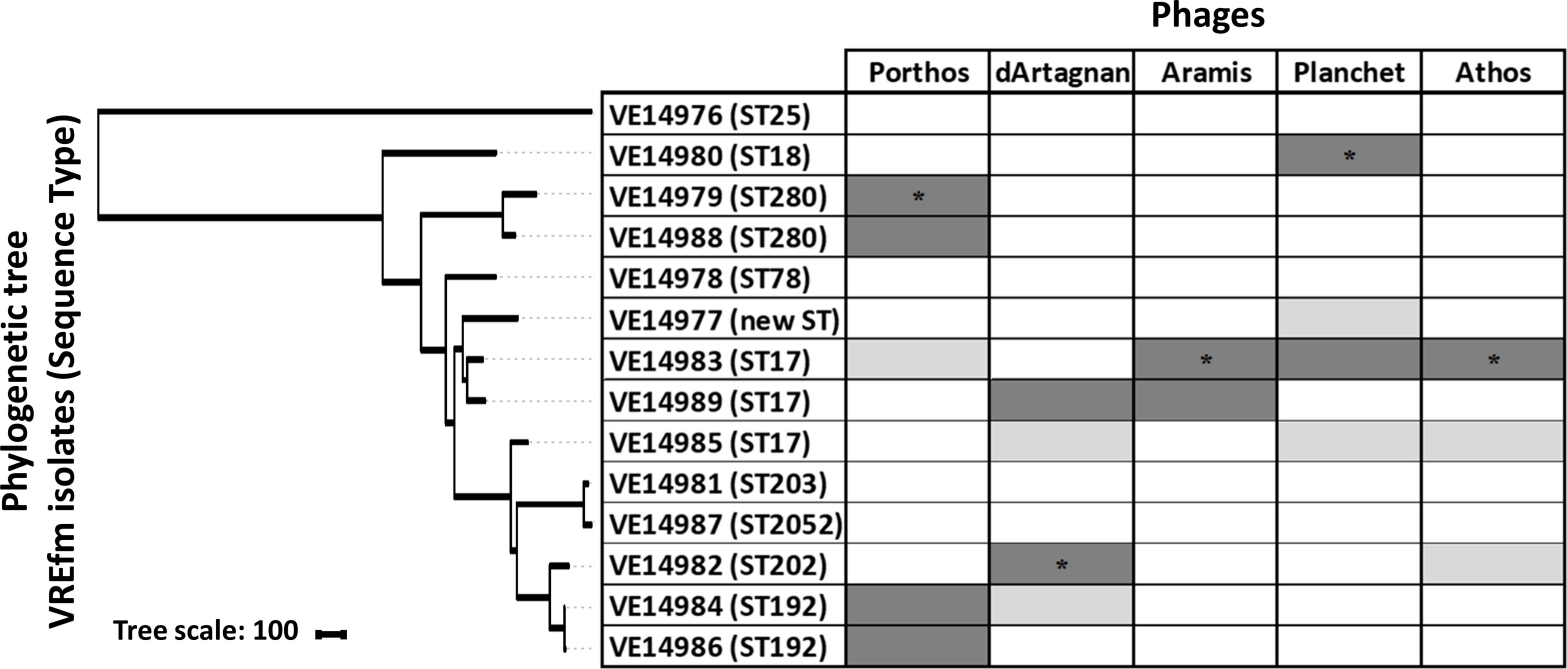
Host ranges of the five phages from the collection with the best and complementary lytic activity. The phylogenetic neighbor-joining tree based on the core genome SNPs was generated with iTOL (31). Host ranges were evaluated by spot assays on the 14 VREfm isolates. Dark gray, strong lytic activity; light gray, weak lytic activity; white, no apparent productive lytic activity. Respective isolation strains are indicated by an asterisk.

A focus was made on the five phages with stronger and complementary lytic activities, which together targeted 10 of the 14 strains (Fig. 1; Table S5). Podophage 16, myophage 4, and siphophages 18 and 6, isolated from wastewater samples, were named after the Alexandre Dumas Musketeers, Athos, Porthos, Aramis, and dArtagnan, respectively. The last phage included was siphophage 27 isolated from a pig fecal virome and was called Planchet, another protagonist of Dumas musketeers’ novels.

**Fig 2:**
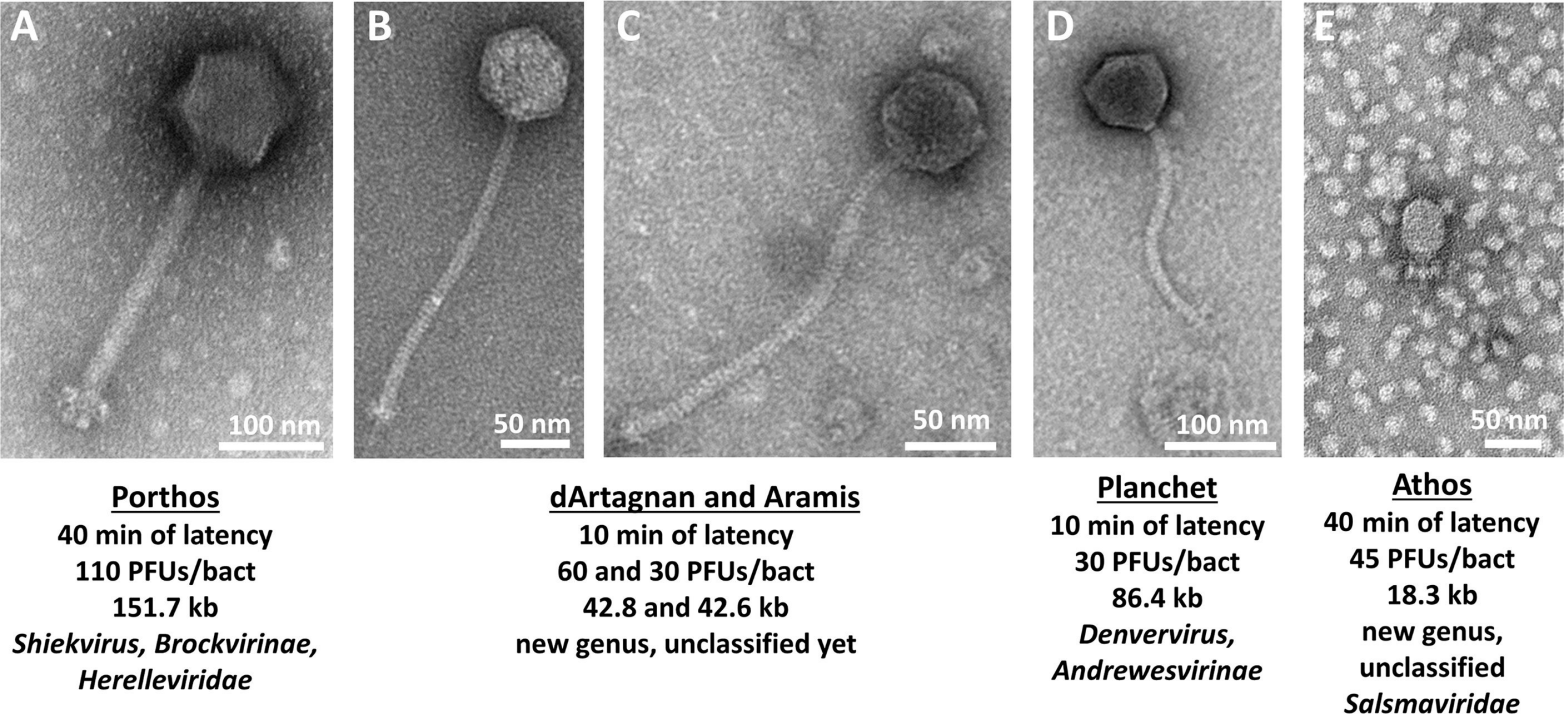
Characteristics of the five phages from the collection with the best and complementary lytic activities. “Identity cards” of Porthos (**A**), dArtagnan (**B**), Aramis (**C**), Planchet (**D**), and Athos (**E**), including their micrographs by transmission electron microscopy, latent periods and burst sizes [PFUs per bacteria (isolation strain)], genome sizes, and taxonomical assignations.

**Fig 3:**
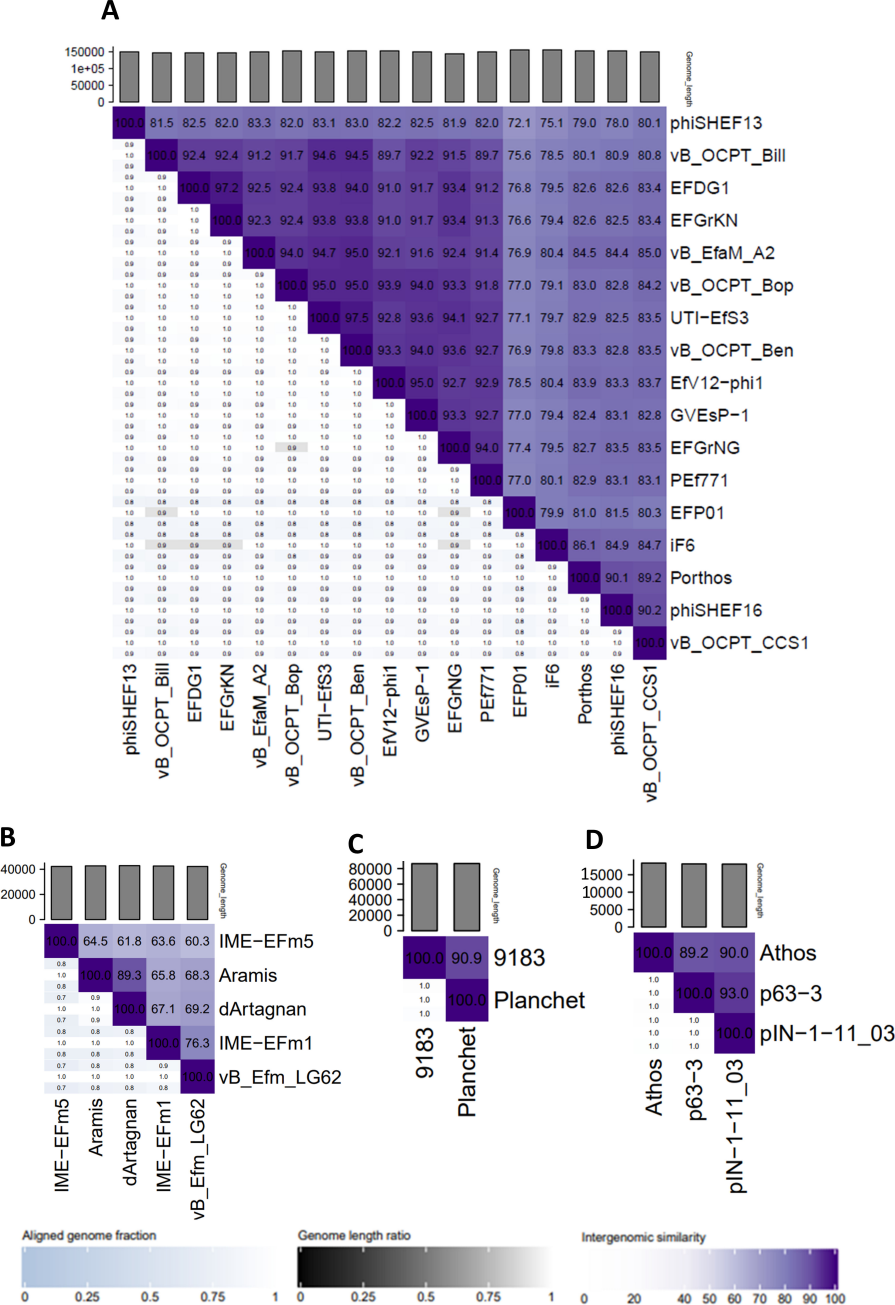
Genomic nucleotide identities between *Enterococcus* phage Porthos (**A**), dArtagnan and Aramis (**B**), Planchet (**C**), or Athos (**D**) and their respective closer relatives. Genbank accession numbers of these genomes are listed in Table S3. The scores and the resulting map were calculated and generated using VIRIDIC tool (47).

**Fig 4:**
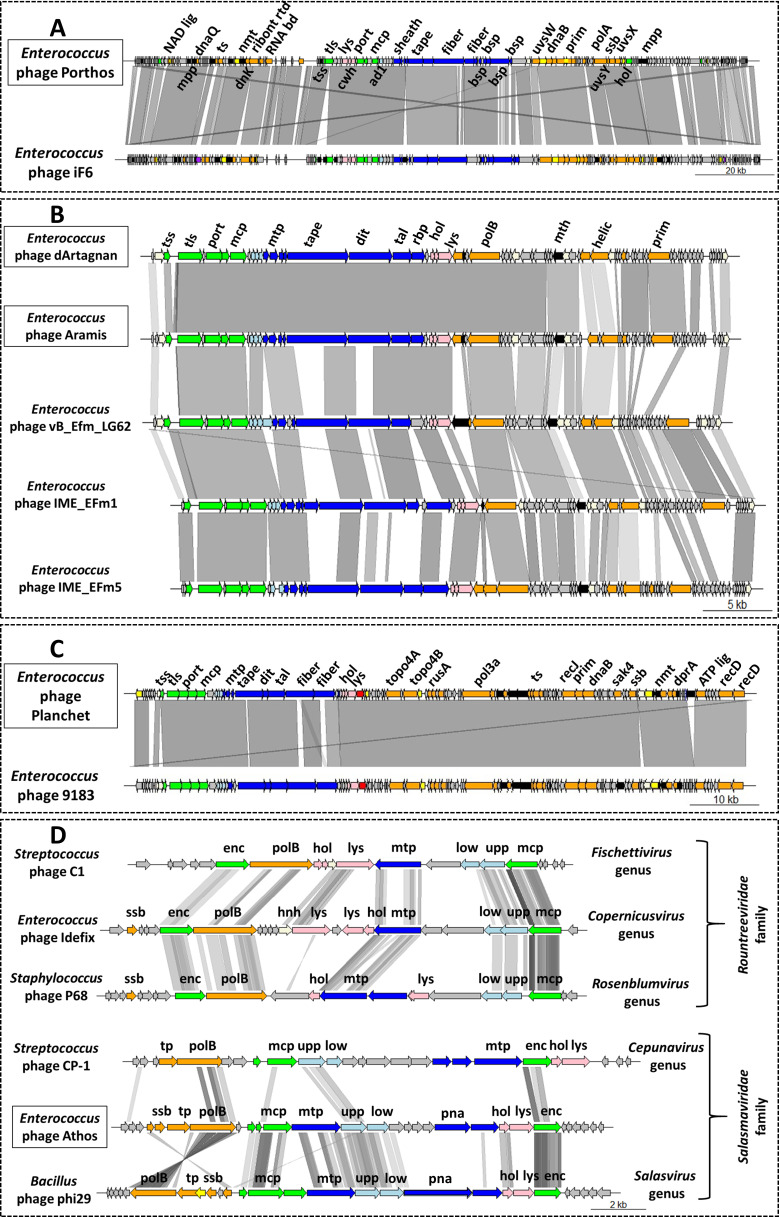
Comparative genomics between *Enterococcus* phage Porthos (**A**), dArtagnan and Aramis (**B**), Planchet (**C**), or Athos (**D**) and their respective closer relatives. Genbank accession numbers of these genomes are listed in Table S3. Genome comparison was performed using genoplotR (46). Shed of gray lines connects phage regions that have BLASTn (**A, B, and C**) or tBLASTx (**D**) identity from 30% to 100% over 100 bp. Gene functions are color coded (and abbreviated): orange, nucleic acid metabolism (NAD lig, NAD-dependent DNA ligase; dnaQ, DnaQ-like 3*'*−5*'* exonuclease; ts, thymidylate synthase; dnK, deoxyadenosine kinase DnK; ribont rdt, component of class III ribonucleotide reductase; RNA bd, RNA-binding protein; uvsW, UvsW-like helicase; dnaB, DnaB-like helicase N terminal domain-containing protein; polA, family A polymerase; polB, family B polymerase; helic, helicase; prim, primase; uvsY, UvsY-like mediator recombination protein; ssb, single-strand DNA-binding protein; uvsX, UvsX-like recombination protein; topo4A and B, DNA topoisomerase 4 subunits A and B; rusA, RusA-like crossover junction endodeoxyribonuclease; pol3a, DNA polymerase III alpha subunit; recJ, RecJ-like single-strand exonuclease; sak4, Sak4-like ssDNA-annealing protein; dprA, DprA-like DNA recombination-mediator protein; ATP lig, ATP-dependent DNA ligase; recD, RecD-like helicase subunit; tp, terminal protein); green, DNA packaging and head (tss and tls, terminase small and large subunits; port, portal; mcp, major capsid protein; enc, encapsidation protein); dark blue, tail [sheath, tail sheath protein; tape, (putative) tail length tape measure protein; fiber, (putative) tail fiber protein; mtp, major tail protein; dit, distal tail protein; tal, tail-associated lysozyme protein; rbp, (putative) receptor-binding protein; bsp, baseplate protein]; light blue, connector (ad1, adaptor protein Ad1; low and upp, lower and upper collar proteins; pna, preneck appendage protein); pink, lysis (lys, endolysin; cwh, cell wall hydrolase; hol, holin); yellow, transcriptional regulation; purple, transposase; red, tyrosine recombinase; white, HNH endonuclease; black, auxiliary metabolism (mpp, metallophosphatase; mth, metallo-hydrolase; nmt, nicotinamide mononucleotide transporter); gray, hypothetical protein.

The myophage Porthos exhibited a strong lytic activity on its isolation strain (VE14979), as well as the other related isolate belonging to the ST280 (VE14988). It also grew efficiently on the two ST192 closely related strains (VE14984 and VE14986) that were phylogenetically distant from the first two targeted strains and had a weak lytic action on a fifth clinical strain (VE1983) from ST17 (Fig. 1). We also tested whether Porthos was able to grow on additional enterococci species. Interestingly, besides infecting *E. faecium,* Porthos formed plaques on three other species: *E. durans* (one of the five tested isolates), *E. hirae* (2/6), and *E. faecalis* (2/9). It did not lyse, however, the single *E. mundtii* isolate tested (Table S7). On its isolation strain, Porthos had a latent period of ~40 min and a burst size ~110 PFUs (Fig. S2A). Porthos virion is composed of a contractile tail ~230 nm in length and ~24 nm in diameter and a polyhedral head ~100 nm in diameter (Fig. 2; Table S6). Porthos has a double-strand linear genome of 151,704 bps with 2,552 bp direct terminal repeats. It represents a new species among the genus *Shiekvirus* (see VIRIDIC comparisons, Fig. 3A) within the subfamily *Brockvirinae* and the family *Herelleviridae* (55). Figure. 4A presents a detailed description of its genome and a comparison with one of its closest relative *Enterococcus* phage iF6 (19). Twenty-four tRNAs were predicted on Porthos as well as 194 ORFs, ~35% of which could be functionally annotated. A third of them were predicted to be involved in virion structure and lysis, a second third in DNA metabolism, and the last fraction in auxiliary metabolic functions.

The two siphophages dArtagnan and Aramis formed clear plaques on two strains, including their respective isolation strains (VE14982 and VE14983), as well as a common third VREfm strain (VE14989). While Aramis was strongly active on two very closely related ST17 isolates (VE1983 and VE1989), dArtagnan only targeted one of those (VE14989) as well as its quite phylogenetically distant isolation strain from ST202 (Fig. 1). In addition, dArtagnan grew on two more isolates: one from the ST17 (VE14985) and one from the ST192 (VE14984) that was phylogenetically close to its isolation strain (Fig. 1). None of them grew on the panel of other enterococci species tested (Table S7). The two phages had similar latent period of ~10 min and respective burst sizes of ~60 and ~30 PFUs (Fig. S2B and C). dArtagnan and Aramis are morphologically very close harboring a flexible tail ~10 nm in diameter and ~230 or 240 nm in length, and a polyhedral head ~63 or 57 nm in diameter (Fig. 2B and C; Table S6). Their genomes are similar in size, 42 kb length, and share 89.3% nucleotide identity over their whole length. A total of 71 and 72 ORFs were predicted in dArtagnan and Aramis genomes, respectively. Both phages are related (61% to 69% nucleotide identity along the entire genomes) to *Enterococcus* phages vB_Efm_LG62 (56), IME-EFm1 (57), and -EFm5 (58) (Fig. 3B and Fig. 4B). A more distant relationship with *Enterococcus* phages 9184 (23) and vB_EfaS-DELF1 (59) is also suggested by ViPTree (Fig. S3). dArtagnan and Aramis represent a new phage genus, with close relationship to the abovementioned phages, for which no taxonomy has yet been proposed.

The third siphophage Planchet was isolated on the ST18 isolate (VE14980), against which it exhibited a strong lytic activity. Three additional VREfm strains were targeted by the phage: one (VE14983) with strong and two (VE14977 and VE14985) with weak lytic activities. This host spectrum covered three distinct STs, including isolates that were quite distant phylogenetically (Fig. 1). Planchet’s burst size was ~30 PFUs with a latent period of ~10 min (Fig. S2D). Its virion harbors a flexible tail ~175 nm in length and ~10 nm in diameter and a polyhedral head ~70 nm in diameter (Fig. 2D; Table S6). It packages a double-strand linear DNA of 86,460 bp, on which 2 tRNAs and 109 ORFs were predicted. Taxonomically, Planchet represents a new species in the *Denvervirus* genus, a genus which also includes phage 9183 (23) within the subfamily *Andrewesvirinae* (Fig. 3C). Both genomes were compared in Fig. 4C. While structural and lysis genes are located in the left part of the genome(s), some auxiliary metabolic genes and numerous DNA metabolism genes are grouped in a divergent operon in the right part of Planchet (and its close relative 9183). Both phages also encode a putative integrase, suggesting they correspond to ex temperate phages.

The podophage Athos had a strong lytic activity on its isolation strain (VE14983, ST17) and a weak lytic activity on two other VREfm strains (VE14985 and VE14982), corresponding to one ST17 isolate and one more phylogenetically distant ST202 isolate (Fig. 1). Outside the *E. faecium* species, this phage also grew on 1/5 *E. durans* strains tested (Table S7). The one-step growth kinetic indicated it had a latent period of ~40 min and a burst size of ~45 PFUs (Fig. S2E). Athos has a short non-contractile tail ~30 nm in length and ~11 nm in diameter and a polyhedral head ~64 nm in length and 42 nm in diameter (Fig. 2E; Table S6). Its slightly elongated head contains a double-strand linear DNA of 18,345 bp. This genome shares ~90% nucleotide identity along its full length with two 18 kb sequences deposited in databases as *Enterococcus* plasmids pIN-1–11_03 and p63-3 (Fig. 3D). Annotation and comparative genomics (Fig. 4D), as well as VIPTree (Fig. S4A) and GRAViTy (Fig. S4B) analyses, revealed Athos corresponds to a new *bona fide* virulent phage genus to which pIN-1–11_03 and p63-3 belong (Fig. S5A). No integrase- or lytic cycle repressor-encoding genes were detected on these genomes, which suggests that they are not temperate phages capable of replicating synchronically with the bacterial host genome as part of a lysogenic cycle. One possibility is that the two phages initially considered as plasmids were actively infecting their respective bacterial strains at the time they were sequenced. Alternatively, Athos-like phages may have the capacity to entertain a pseudolysogenic (60) or carrier state (61) relationship with certain hosts. Athos precisely encodes 29 ORFs, 15 of which were assigned a putative function. Its replication module includes a histone-like protein, a single-strand DNA-binding protein (Ssb), a terminal protein (Tp), and a family-B DNA polymerase belonging to the “protein priming” subfamily (PolB). This organization is characteristic of members of the former *Picovirinae* subfamily (62). To illustrate the diversity of these small virulent podophages, which have recently been reclassified, their genomic comparison was represented in Fig. 4D. *Streptococcus* phage C1 (63) and *Staphylococcus* phage P68 (64), respectively, belonging to the *Fischettivirus* and *Rosenblumvirus* genera are now delimiting the *Rountreeviridae* family, which also includes various *Enterococcus* phages (53) belonging to the *Copernicusvirus* genus. Athos synteny as well as its proportion of shared proteins with other phages (Fig. 4D; Fig. S4) placed it in a different family, the *Salasmaviridae,* together with the reference *Bacillus* phage phi29 (65) (*Salasvirus* genus) and the *Streptococcus* phage Cp-1 (66) (*Cepunavirus* genus). Of note, Athos genetic organization was also close to that of *Actinomyces* phage Av-1 (67) belonging to the *Dybvigvirus* genus and currently unclassified at subfamily/family levels (Fig. S5B).

### Phage combinations

Phage combinations were chosen to provide ample “breadth” and “depth” of activity. The breadth of a cocktail is the number of targeted bacterial isolates, while its depth reflects its ability to reduce the bacterial capacity to evolve phage resistance. A simple way to achieve a good depth is to combine phages targeting different receptors on the bacterial surface (68, 69).

Based on individual phage host spectra, dArtagnan, Porthos, and Planchet were expected to have collectively the broader spectrum of activity against the VRE tested (Table S8). To get an overview of the depth of activity, we isolated and characterized spontaneous phage-resistant mutants derived from VE14983 on which four out of the five phages grew and investigated the cross-resistance of the mutants to each of these phages.

Four mutants resistant to Aramis (Aramis-R1, R2, R3, and R4) and four mutants resistant to Athos (Athos-R1, R2, R3, and R4) were obtained starting from VE14983. No visible plaques were produced on the resistant mutants when infected with Aramis or Athos (Table S9). Adsorption assays indicated that Aramis exhibited an ~4-to-10-fold reduced adsorption efficiency on its four resistant mutants (Fig. S6A), while Athos had an ~4.5-to-15-fold reduced adsorption efficiency on Athos-R1, R2, and R3 and a milder adsorption defect on Athos-R4 (Fig. S6B). We therefore concluded resistance to Aramis and Athos was due to adsorption defects. Sequencing of the eight resistant strains revealed in all cases a point mutation in genes of the *epa* locus and mostly of its variable part (Table S10). *E. faecium* exhibits an *epa* locus organized similarly to that of *E. faecalis* (70), in which the variable part of the locus (as well as the downstream *epaR* gene) is involved in cell wall teichoic acid biosynthesis (71). Mutations in this region prevent the adsorption of various phages in *E. faecalis* (53, 72) and *E. faecium* (23). No plasmid being available to attempt complementation studies, we can only suggest here, thanks to the common set of mutations in *epa*, that teichoic acids are necessary for the binding of phage Aramis and Athos to the cell surface.

We next controlled the cross-resistance of the mutants to each of the other phage. The profiles were almost identical for the two phages: all strains resisting to Aramis also strongly resisted to Athos, and the converse is true, except that the Athos-R4 strain was only partially resistant to Aramis (Table S9).

In contrast, the myovirus Porthos was able to grow as well on VE14983 as on the *epa* mutant derivatives resisting to Aramis or Athos (Table S9), suggesting that Porthos receptor is different from teichoic acids. Indeed, when a similar analysis of resistant colonies was performed with Porthos on its VE14979 isolation strain, none of the four mutants tested were adsorption mutants (Fig. S6C). The Porthos receptor remains therefore unknown at this stage. The siphophage Planchet also grew as well on the Aramis-resistant mutants as on the VE14983 strain. This was also the case on Athos-R3 and -R4, while Athos-R1 and -R2 only partially resisted to Planchet (Table S9). Although previous results indicated 9183, a phage from the same genus as Planchet, used teichoic acids as receptor (23), Planchet was not, or only partially, impacted by the *epa* mutations found in the VE14983 derivatives resisting to Aramis or Athos.

Following these analyses, we decided to mix dArtagnan, Porthos, Planchet, and Aramis, excluding Athos, as the latter was only strongly active on its isolation strain and was clearly susceptible to target the same type of receptor as Aramis.

Exposure to phage selective pressure favoring mutations in the variable part of *epa* locus contributes to the loss of vancomycin resistance in *E. faecalis* (21) and to an enhanced susceptibility of an *E. faecium* laboratory strain to β-lactams (23). We consequently performed AST for the four Aramis- and Athos-resistant, as well as one Porthos-resistant mutants compared to their parental strains to determine whether phage resistance also altered antimicrobial susceptibility of an *E. faecium* CC17 clinical isolate. All Aramis- and Athos-resistant mutants interestingly showed a significant decrease (4- to 16-fold) in MIC of teicoplanin. In addition, some of the mutants (Athos-R1 to R3 and Aramis-R1) exhibited a 4-to-8-fold reduction in MIC of daptomycin (Table S11). MIC of lipoglycopeptides was also slightly but significantly altered for Athos-R3 (telavancin) and Aramis-R2 (telavancin and dalbavancin) (Table S11). It may be, therefore, that concurrent treatment with antibiotics and the phages studied here provide synergistic effects. Synergistic effects between phages and β-lactam/daptomycin were already observed *in vitro* or *ex vivo* for some *E. faecium* isolates (22–24). Clinically, a concurrent treatment with traditional antibiotics (that alone had failed to eradicate recurrent *E. faecium* bacteremia) with phages temporarily suppressed the episodic bloodstream infections and lowered the intestinal *E. faecium* load of a patient (73). These data underline the clinical relevance in pursuing *in vitro* and pre-clinical studies assessing the possibilities offered by phage and antibiotic combinations to fight enterococci infections (73). We tested whether the combination of phages and teicoplanin reduced bacterial overgrowth. For this, we plated Aramis with its isolation strain (VE14983) at MOI ~50 in the presence of the antibiotic at the MIC and did not observe any bacterial-resistant colonies after 20 h of incubation. In contrast, spontaneous colonies arose at frequencies of ~10^−5^ when the phage or the antibiotic was used separately on VE14983 at the same respective concentrations (Table S12). These results encourage further investigations to evaluate whether a phage/teicoplanin combination could be therapeutically exploited.

### Extending host spectra by phage training

We next attempted to extend the host range of dArtagnan, Porthos, Planchet, and Aramis, using the Appelman protocol. The inclusion of Aramis in the cocktail was (also) due to its genetic relatedness with dArtagnan, opening the possibility of efficient homologous recombination. Eight VREfm resistant to two, three, or four of the phages were used as development strains for the cocktail (Fig. 1; Table S13). Each strain was cultivated and subjected separately to the phage cocktail. Fifteen successive passages of the initial phage cocktail, containing ~10^8^ PFUs/mL of each phage, significantly improved its lytic activity, as seven of the eight strains were lysed, of which five with the minimal phage dose (Fig. S7). Concerning strains VE14978 and VE14981 that were totally resistant to all phages at the onset, the first one harbored an intermediary susceptibility, while the second largely remained resistant to the evolved cocktail.

To investigate which phage(s) had contributed to the extended host range, 18 phages were isolated and purified on 7 of the development strains (Table S13). The 18 phages exhibited 11 distinct profiles, killing up to 5 strains and encompassing together the 8 development strains (Table S14). In comparison, the four initial phages targeted between one and three strains and covered six out of the eight strains together. Five evolved phages, including K.35.2, K36.1, K36.3, and K.41.1 with broader host ranges and K38.2 with a narrow spectrum but able to grow on the phage-cocktail refractory VE14981 strain (Table S14), were further characterized by testing their lytic activity on the six remaining VREfm strains from our set (on which the phages were not trained). Spot assays indicated that K35.2, K36.1, K36.3, and K41.1 indeed exhibited extended host ranges, being able to lyse 9 to 10 out of the 14 tested strains. This represented approximately twice as much strains as the more efficient native phage (Porthos) and included two strains that were not sensitive to any of the initial phages (Table S15). Among them, K35.2 was the most efficient, as it showed a strong lytic activity on eight of its nine target strains (Table S15). Its host spectrum, like for K41.1, has not been extended to other enterococci species tested (Table S16). The last of the five selected evolved phages, K38.2, lysed only three strains (Table S15).

### Comparative genomic analysis of evolved and parental phages

**Fig 5:**
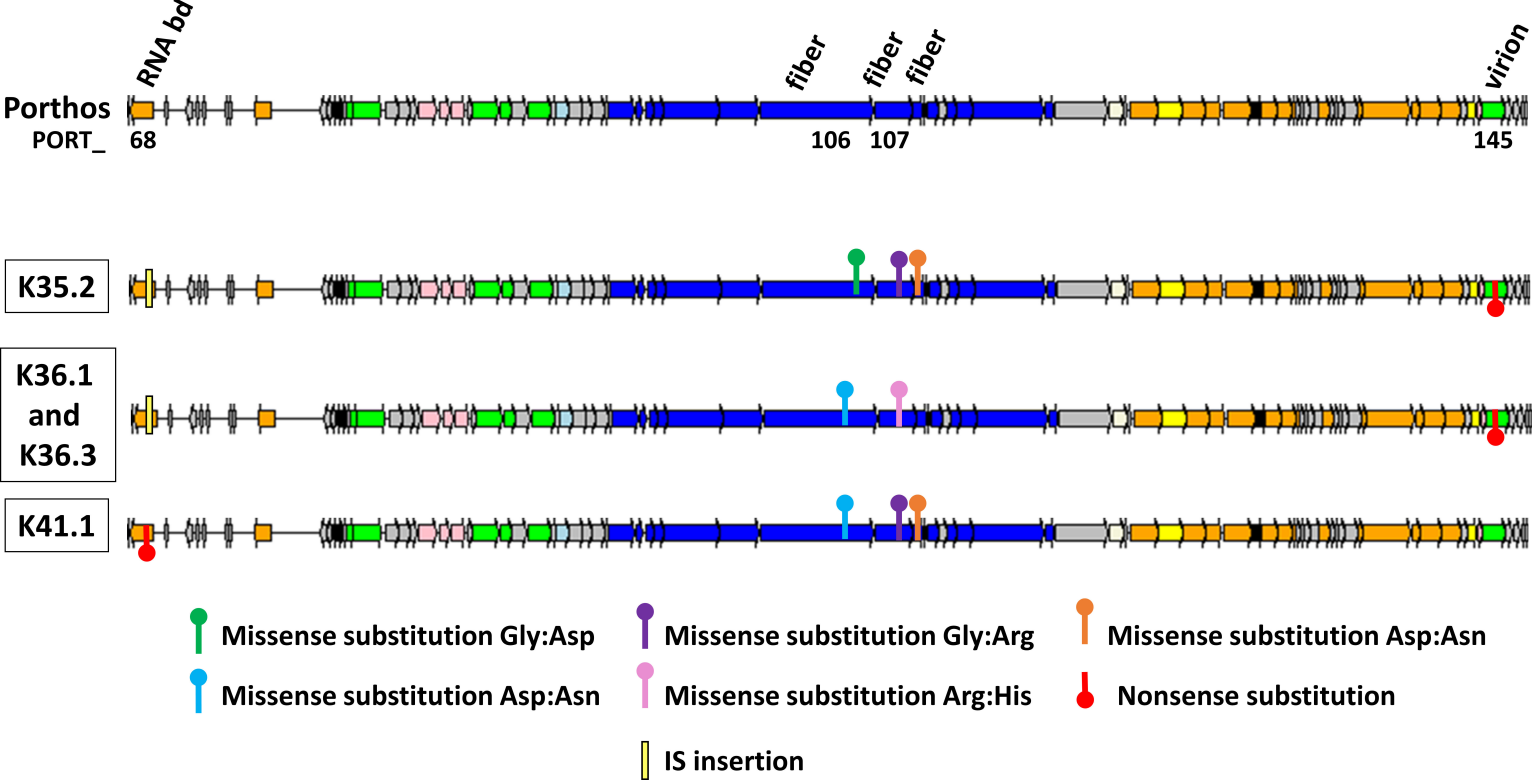
Most significant mutational events detected in K35.2, K36.1, K36.3, and K41.1, which all were derived from *Enterococcus* phage Porthos. Only the relevant part of the genomes with most mutations were shown here using genoplotR (46). The complete genome of Porthos is shown in Fig. 3A, and all base substitution mutations and small indels detected using breseq (52) are detailed in Fig. S8. The common missenses substitutions detected in PORT_107- and PORT_124-encoding genes for the four evolved phages are not represented here. IS insertion was detected following global alignments of the evolved phages against Porthos. Gene functions are color coded: orange, nucleic acid metabolism; green, DNA packaging and head; dark blue, tail; light blue, connector; pink, lysis; yellow, transcriptional regulation; white, HNH endonuclease; black, auxiliary metabolism; gray, hypothetical proteins. Selected genes are annotated and abbreviated; RNA bd, RNA-binding protein; fiber, (putative) tail fiber protein; virion, virion structural protein.

**Fig 6:**
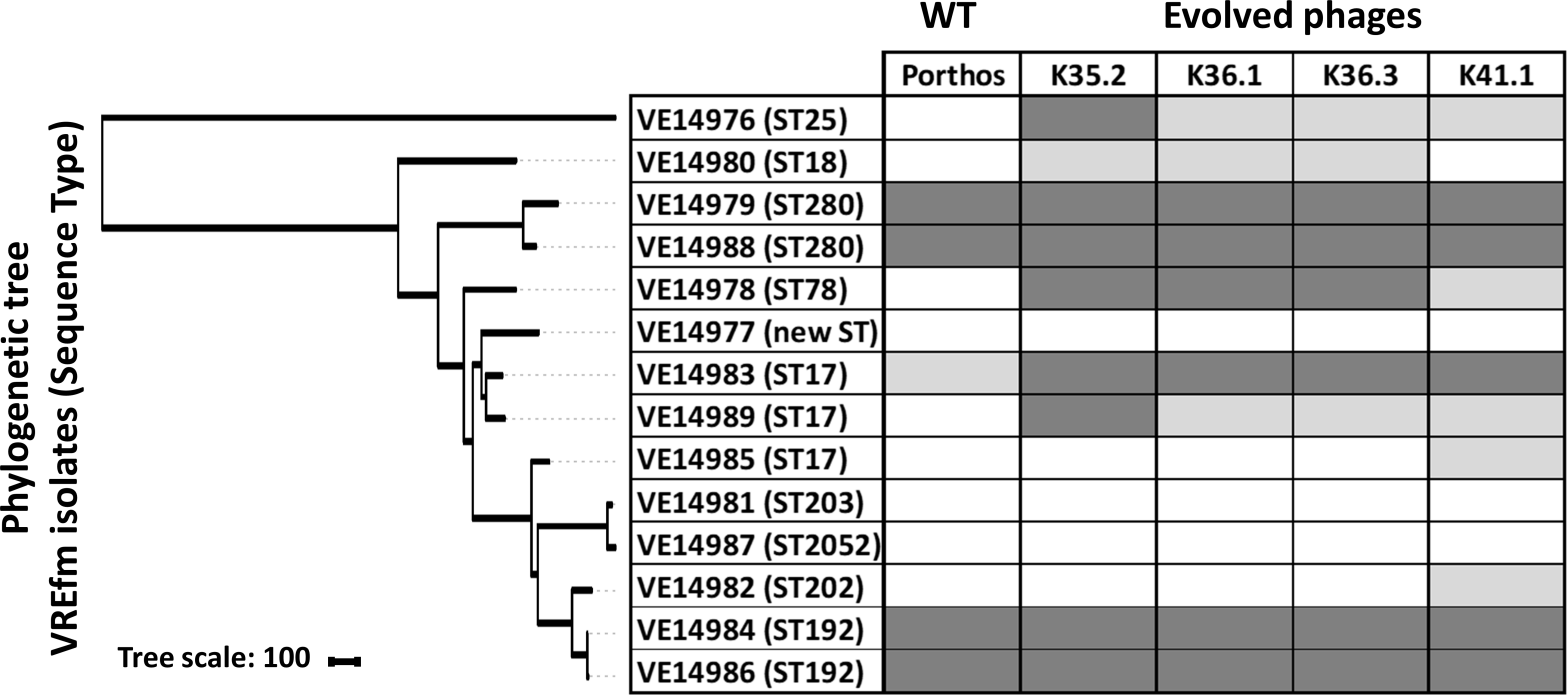
Comparison between the host range of *Enterococcus* phage Porthos and those of the four evolved phages from which they were derived. The phylogenetic neighbor-joining tree based on the core genome SNPs was generated with iTOL (31). Host ranges were evaluated by spot assays on the 14 VREfm isolates. Dark gray, strong lytic activity; light gray, weak lytic activity; white, no apparent productive lytic activity.

The sequencing and assembly of the evolved phages revealed that four of them, K35.2, K36.1, K36.3, and K41.1, were derived from Porthos (Fig. 5) and contained between seven and nine point mutations (Fig. S8). Two mutations were shared by all mutants, a missense substitution in a putative antisigma factor PORT_124- and a putative tail fiber PORT_107-encoding genes. Additional and differing missense substitutions were notably found in the tail fiber region. For instance, the more efficient mutant K35.2 [with a strong lytic activity on 8/9 susceptible strains belonging to six distinct STs (Fig. 6)] harbored a specific mutation in the tail fiber gene PORT_106 and shared two others with K41.1 in other putative tail fiber genes PORT_107 and PORT_108 (Fig. 5; Fig. S8). K41.1 [active on 10 isolates from 6 different STs (Fig. 6)] harbored a mix of the point mutations detected in the tail fiber genes of the other mutants (Fig. 5; Fig. S8). Intriguingly, the coding sequence of a putative RNA-binding protein (PORT_ 68) was disrupted by an IS*200*/IS*605* insertion sequence in K35.2, K36.1, and K36.3, while a nonsense substitution interrupted the coding sequence in K41.1 (Fig. 5; Fig. S8). We have no clue of the function of this gene. The expected involvement of tail fiber proteins in phage adsorption (74, 75) led us to compare the adsorption efficiencies between Porthos and mutant K35.2. After a 10-min incubation at 37°C, K35.2 adsorption (~75%) was indeed significantly better than that of Porthos (~45%) on two tested strains (VE14978 and VE14989) sensitive to the evolved phage but resisting to Porthos (Fig. S9). We verified that both phages had an equal capacity to adsorb to strain VE14984, in which they both grow well.

**Fig 7:**
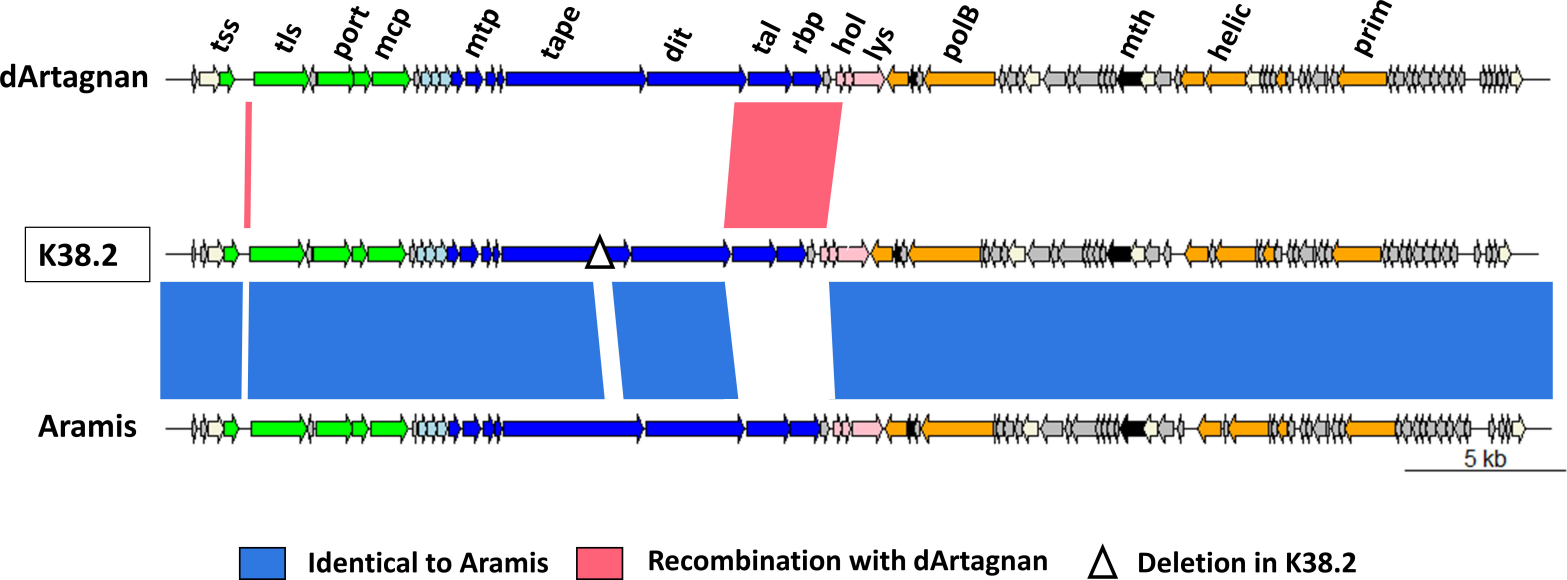
Mutational events detected in K38.2, which was mainly derived from *Enterococcus* phage Aramis having recombined with the closely related *Enterococcus* phage dArtagnan. The three genomes were represented using genoplotR (46). Gene functions are color coded (and abbreviated): orange, nucleic acid metabolism (polB, B family polymerase; helic, helicase; prim, primase); green, DNA packaging and head (tss and tls, terminase small and large subunits; port, portal; mcp, major capsid protein); dark blue, tail [mtp, major tail protein, tape, tail length tape measure protein; dit, distal tail protein; tal, tail-associated lysozyme protein, rbp, (putative) receptor-binding protein]; light blue, connector; pink, lysis (lys, endolysin; hol, holin); white, HNH endonuclease; black, auxiliary metabolism (mth, metallo-hydrolase); gray, hypothetical proteins. The mutational events are indicated below.

**Fig 8:**
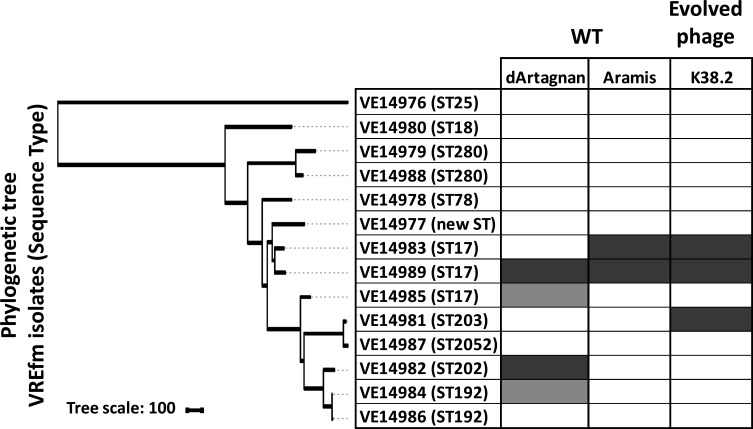
Comparison of the host ranges of *Enterococcus* phages dArtagnan and Aramis with those of K38.2 from which it was derived. The phylogenetic neighbor-joining tree based on the core genome SNPs was generated with iTOL (31). Host ranges were evaluated by spot assays on the 14 VREfm isolates. Dark gray, strong lytic activity; light gray, weak lytic activity; white, no apparent productive lytic activity.

Finally, the last selected mutant, K38.2, corresponded to a hybrid between phages dArtagnan and Aramis, with at least two crossovers between them. The evolved phage is mainly derived from Aramis, with two parts of its genome identical to dArtagnan (Fig. 7). The first one corresponds to the small intergenic region between the terminase subunit-encoding genes and the second to an ~3 kb region extending from a part of the tail to a part of the lysis modules. The region notably includes genes coding the distal tail protein (Dit), the tail-associated lysozyme protein (Tal), and the putative receptor-binding protein (RBP). Additionally, K38.2 harbors a 366-bp deletion in the gene for the tail length tape measure protein (Fig. 7). K38.2 targeted the two strains susceptible to Aramis, as well as a new one VE14981 (Fig. 8).

## DISCUSSION

The host range is a key property in phage therapy, and isolated phages are commonly reported to have a narrow host range of related strains (76). While it is preferable to use a phage whose host range is limited to a single bacterial species to prevent collateral effects against other species of the considered microbiota, a phage that is able to lyse multiple strains within the targeted species is desirable. Avoiding the need to screen phages against the specific pathogen responsible for the infection would save valuable time in terms of treatment (76, 77). Several methods have been developed to select natural phages with extended host range in term of strains within a target species, or beyond (76, 77). Despite its common use in IBMV and in several other labs from the Eastern Europe, the Appelmans protocol was only recently applied in Western studies to extend *Pseudomonas aeruginosa* phages host spectra (26, 27).

Here, we studied in detail five virulent phages against CC17 VREfm isolates, which represent a growing clinical problem worldwide, and used the Appelmans protocol to enlarge the host ranges of four of these phages. Eight development VREfm strains were chosen to evolve the phage cocktail initially composed of (i) the myophage Porthos that exhibited the broader plaquing host range, (ii) the two very close siphophages dArtagnan and Aramis, (iii) and the completely different siphophage Planchet. The four-phage combination should, in theory, have infected six of the eight development strains, but intriguingly, two strains susceptible to Porthos, and one to Aramis and dArtagnan, were not lysed following the first passage. This may be due to antagonism of the phages initially combined in the cocktail toward some strains. Another study recently reported that the sum of the lytic activities of individual phages targeting *Escherichia coli* strain O157:H7 did not reflect the lytic activity of all phages, with some phage combinations being synergistic, neutral, or antagonistic (78). In our experiment, at the end of the 15th passage, one of the eight strains remained refractory to the evolved phage cocktail. This is quite similar to the endpoint of the original Appelmans protocol, which is reached when ≥80% of the development strains have lysed up to the maximal dilution rate (27).

Four of the five phages isolated from the evolved cocktail (K35.2, K36.1, K36.3, and K41.1) exhibited extended host ranges with approximately twice as much VREfm-susceptible strains as Porthos, from which they all derive. In addition to extending their host range on five of the eight development strains, the evolved phages remained active on the three other VREfm strains targeted by Porthos and became active on one or two new strains, depending on the phage mutants. This brings the total number of VREfm strains susceptible to these evolved phages to 9 or 10 out of the 14 VREfm strains. The 9 or 10 strains belong to 6 distinct STs out of the 10 tested here. As Porthos is able to grow on few other enterococci species, 2 of its evolved derivatives were tested on the same set of 21 enterococcal isolates belonging to species related to *E. faecium (E. durans*, *E. mundtii*, and *E. hirae*) or to the clinically relevant species *E. faecalis*. However, the interspecies host range of the mutants was not broader than that of Porthos. This shows that the Appelmans protocol is a valuable method for generating virulent phages with broader host spectra within a given *Enterococcus* species.

Point mutations, rather than recombination, seemed to be the drivers of the evolution for the four Porthos-derived myophages. Between three and four SNPs per phage are in the tail fiber region, and one of them is common to all four. The mutation profiles are congruent with the various host spectra: K36.1 and K36.3 are slightly different but contain the same mutations in the tail fiber region and have identical infection profiles. K35.2 has a different set of tail fiber mutations and a differing host spectrum. Finally, K41.1, which has a combination of the two latter (K36.1/K36.3 and K35.2) tail region mutations, has its own host range. Tail fiber proteins usually function as RBPs and mediate the adsorption for many myophages infecting gram-negative or -positive bacteria (74, 75). RBP-encoding genes were also demonstrated as evolving faster than other genes from phage genomes, given that they can induce favorable host spectrum modifications (79–81). K35.2 adsorbs better than its parental phage Porthos to two tested VREfm strains that became sensitive to the evolved phage. However, given the partial capacity of Porthos wild-type to adsorb to these strains, mutations in the tail fiber proteins may not be sufficient to fully explain the expanded host ranges of the evolved phages.

An intriguing common point between the four Porthos-derived phages is the interruption of a gene encoding a putative RNA-binding protein. For three of them, disruption was through the insertion of an IS from the IS*200*/IS*605* family. As transposable elements, IS can move by themselves within and between the different types of DNA molecules contained in a bacterium, but they need conjugative elements or transducible phages for intercellular movement (82). Nosocomial and virulent *E. faecalis* and *E. faecium* strains often contain multiple IS elements (83). Their patho-adaptation is proposed to be due in part to IS from the IS*256* family, via gene inactivation and recombination events. In the *E. faecalis* strain V583, IS*256* activity was shown to drive genome diversification in response to specific antibiotic use or phage predation (84). Our results underline that IS*200*/IS*605* also seems able to diversify the genome of an invading virulent phage. Whereas IS transposition should be detrimental to the infecting phage in general (and could even be seen as a bacterial way of defense), the present IS-mediated interruption does not seem to affect the fitness of our evolved phages. We can even wonder if the disruption of the predicted RNA-binding protein-encoding gene (which function is unknown) is not favorable to the lytic capacity of the phages, given that a nonsense mutation is detected in the same gene for the fourth Porthos mutant.

A limitation of this study is that a single evolution experiment, i.e., a single replicate of successive 15 passages, was performed. Analysis of mutations occurring in other genetic lineages under the same constraints would help clarify mutations favoring Porthos host range extension. Porthos belongs to the *Brockvirinae* subfamily that was recently shown to be largely distributed in human and animal fecal metagenomes. *Brockvirinae* also enter into the composition of two IBMV phage cocktails used in the treatment of intestinal disorders (17). Porthos precisely belongs to the *Shiekvirus* genus. One of these phages, If6 (19), was recently reported for the promising activity of its endolysin. Two others, EFGrKN and EFGrNG (85), were recently successfully used in a preparation to treat a VRE liver infection of a 1-year child (25), indicating the therapeutic potential of this phage genus.

Porthos exhibits the broader productive host spectrum among our phage collection and has also the capacity to grow on multiple enterococcal species, making it probably a “broad host range” phage, although no strict definition of this character is at hand (76). Two other *Shiekvirus*, vB_OCPT_Ben and _Bop, were reported to have bactericidal activity on a broad range of both *E. faecium* and *E. faecalis* strains, including VRE (17). This characteristic might be related to the bacterial receptor(s) used by these myophages. At present, however, no real clues about Porthos or any other *Shiekvirus* receptor(s) are available. While myophages of the *Herelleviridae* (family to which belong the *Shiekvirus*), such as *Staphylococcus* phages K-like and *Bacillus* phage SPO1, bind the cell wall teichoic acid polymers (86–88), it seems that the bacterial receptor(s) used by *Shiekvirus* could be different. Indeed, Porthos was insensitive to bacterial mutations that putatively blocked Athos and Aramis adsorption and were located in the variable part of the *epa* locus. Mutations in exopolysaccharide capsular *yqw* locus genes were reported following a co-evolution experiment between an *E. faecium* strain and the *Shiekvirus* EfV12-phi1 (89), but then this type of mutation could not be attributed to *Shiekvirus* phages selective pressure in another study (17). If *Shiekvirus* phages use two receptors, this may explain the difficulties encountered in isolating spontaneous adsorption mutants. In this case, Porthos-resistant mutants are most likely mutated in a single gene required in the downstream steps of the adsorption process, leaving the bacterial receptor(s) unexplored and resistant to investigation. Consistently, another gene that was shown to mutate in *E. faecium* when co-evolving with EfV12-phi1 was *rpoC*, which was proposed as a bacterial resistance mechanism by disrupting RNA polymerase activity and thus preventing continuation of the phage lytic cycle after injection (89).

While point mutations were selected during Porthos evolution for host range extension, recombination events also occurred during our evolution experiment. The last phage isolated was the result of two recombination events between Aramis and dArtagnan, which share an average of 89% nucleotide identity. One of these crossovers concerns a region including three genes, coding the tail proteins Dit, Tal, and the putative RBP. This phage, which is mostly composed of Aramis, also harbors a 366-bp deletion in the tail length tape measure protein-encoding gene. All these mutations could have contributed to slightly extend its host spectrum to an additional VREfm strain insensitive to all our other phages. In our experiments, recombination seemed to have been less impactful than during those reported by Burrowes and colleagues. From their 30 round-directed *in vitro* training of a 3-siphophage cocktail on a set of 10 *P*. *aeruginosa* strains, they obtained an evolved phage that was the result of multiple recombinational events between two (of the three) initial phages [sharing 99% of nucleotide identity, (27)]. The balance between point mutation and recombination will greatly depend on input phages (co-infection possibilities, genomic similarities, burst-sizes …) and targeted hosts.

To conclude, our Appelmans experiment permitted to boost a phage cocktail and isolate phages harboring enlarged host ranges within the *E. faecium* species, with a myophage from the *Shiekvirus* genus that was now able to kill up to 10 of the 14 initial VREfm strains while barely infecting closely related species. These results are encouraging and reinforce the notion that *Shiekvirus* and more largely the subfamily of *Brockvirinae* represent promising therapeutic tools against VRE infections. This also illustrates the Appelmans approach, based on selection applied on gene variation, thanks to spontaneous mutations and recombination, is an elegant and rapid way to enlarge host range of virulent phages and fight against MDR infections.

## Data Availability

Raw read data of the 14 VREfm isolates (from VE14976 to VE14989 [Table S1]) were deposited in the European Nucleotide Archive at EMBL-EBI under the accession numbers ERR12663061, ERR12663062, ERR12663063, ERR12663064, ERR12663065, ERR12663066, ERR12663098, ERR12509735, ERR12663099, ERR12663100, ERR12663101, ERR12663102, ERR12663103, and ERR12663104, respectively (study: PRJEB41784). Athos, Porthos, Aramis, dArtagnan, and Planchet genome sequences have been deposited in the European Nucleotide Archive at EMBL-EBI under the accession numbers LR990834, LR990835, LR990833, LR991625, and OX422066, respectively (study: PRJEB41784). The raw read data for the eight genomes of Aramis-R1 to -R4 and Athos-R1 to -R4 were deposited in the European Nucleotide Archive at EMBL-EBI under the accession numbers ERR12454984, ERR12455020, ERR12455021, ERR12476512, ERR12476514, ERR12476515, ERR12476517, and ERR12476519, respectively (study: PRJEB41784).

## SUPPLEMENTAL MATERIAL

Supplemental figures and tables aac.01439-23-s0001.pdf Figures S1 to S9; Tables S1 to S16.

ASM does not own the copyrights to Supplemental Material that may be linked to, or accessed through, an article. The authors have granted ASM a non-exclusive, world-wide license to publish the Supplemental Material files. Please contact the corresponding author directly for reuse.
