# Supplementary material for: The virtue of training: extending phage host spectra against vancomycin-resistant Enterococcus faecium strains using the Appelmans method
Source: Antimicrob Agents Chemother. 2024 Apr 9;68(5):e01439-23. doi: 10.1128/aac.01439-23 (PMC11210271; doi:10.1128/aac.01439-23)
Supplement: Supplemental figures and tables — Figures S1 to S9; Tables S1 to S16. [file aac.01439-23-s0001.pdf]

Supplemental figures

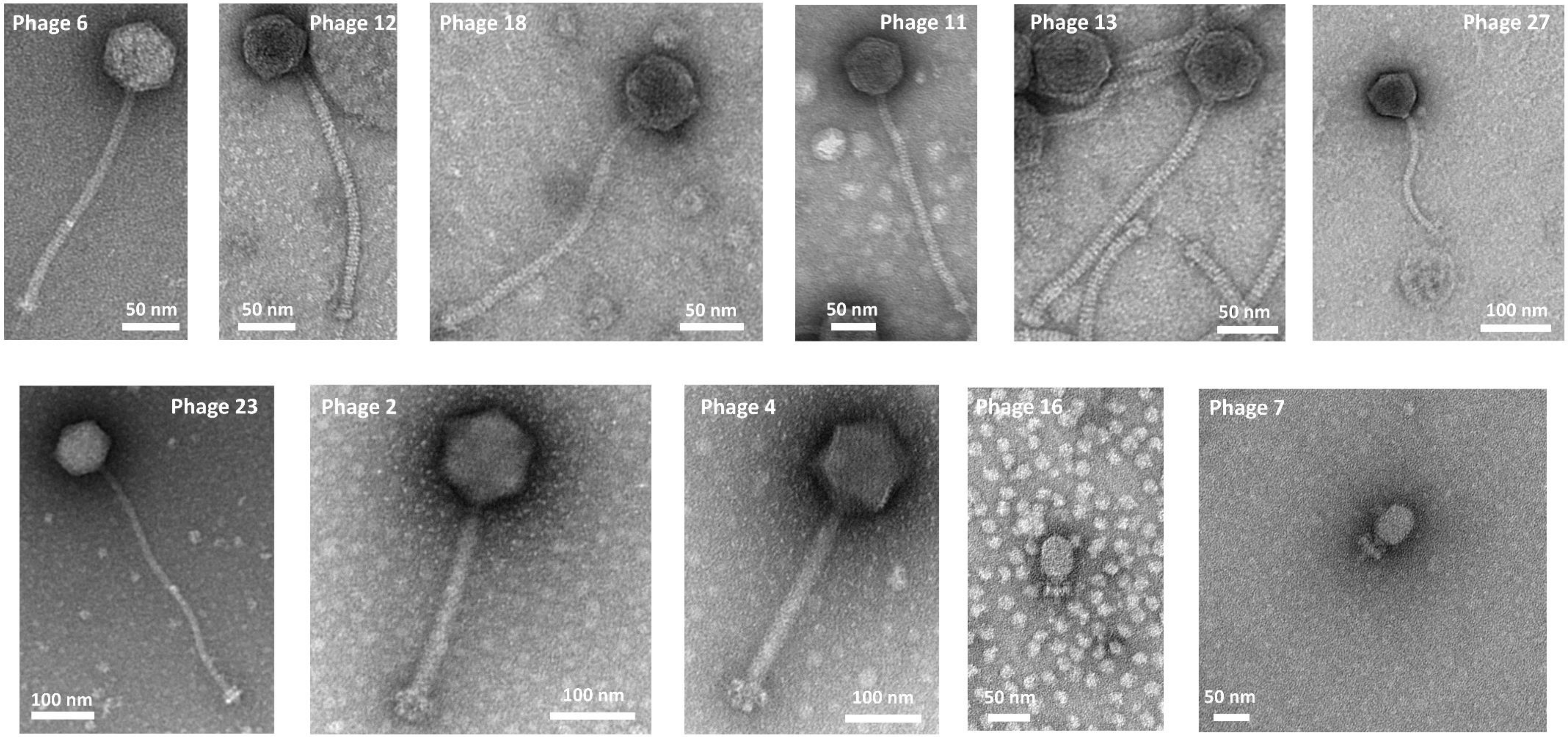

Figure S1. Examination of phages by transmission electron microscopy. All phages negatively stained with 1 % uranyl acetate belonged to the class of *Caudoviricetes*. Phage 6, phage 12, phage 18, phage 11, phage 13, phage 27 and phage 23 were siphophages. Phage 2 and phage 4 were myophages. Phage 16 and phage 7 were podophages.

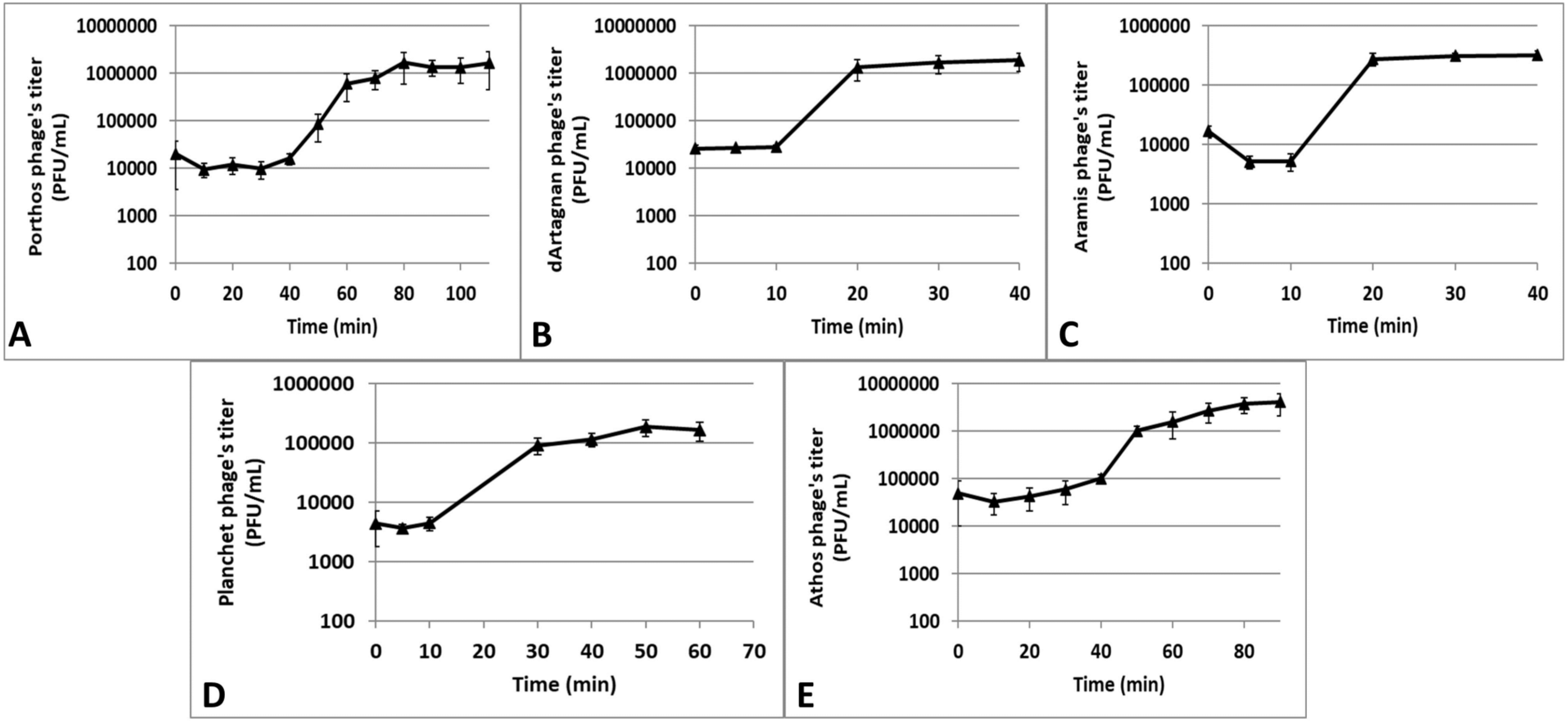

Figure S2 : One step growth kinetics of phages Porthos (A), dArtagnan (B), Aramis (C), Planchet (D) and Athos (E) on their respective isolation strains**.** For each kinetic, the values indicate the means and standard deviations of three independent experiments.

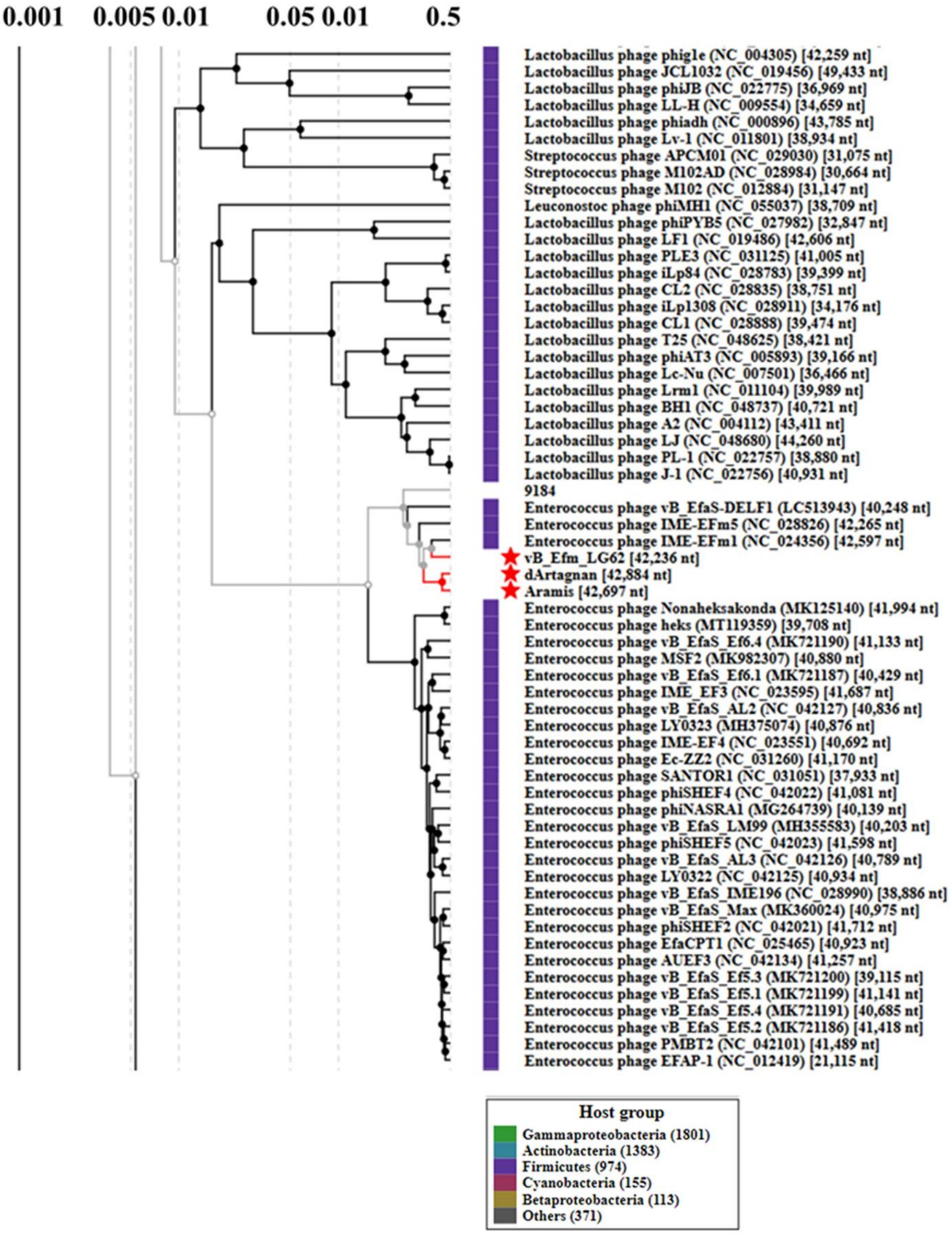

Figure S3. Placement of *Enterococcus* phages dArtagnan and Aramis in the dendrogram generated by whole proteome tBLASTx similarity with VIPTree (49). The relevant region of the tree where the siphophages are positioned is shown, and dArtagnan, Aramis and vB_Efm_LG62 (56) are labeled with red stars.

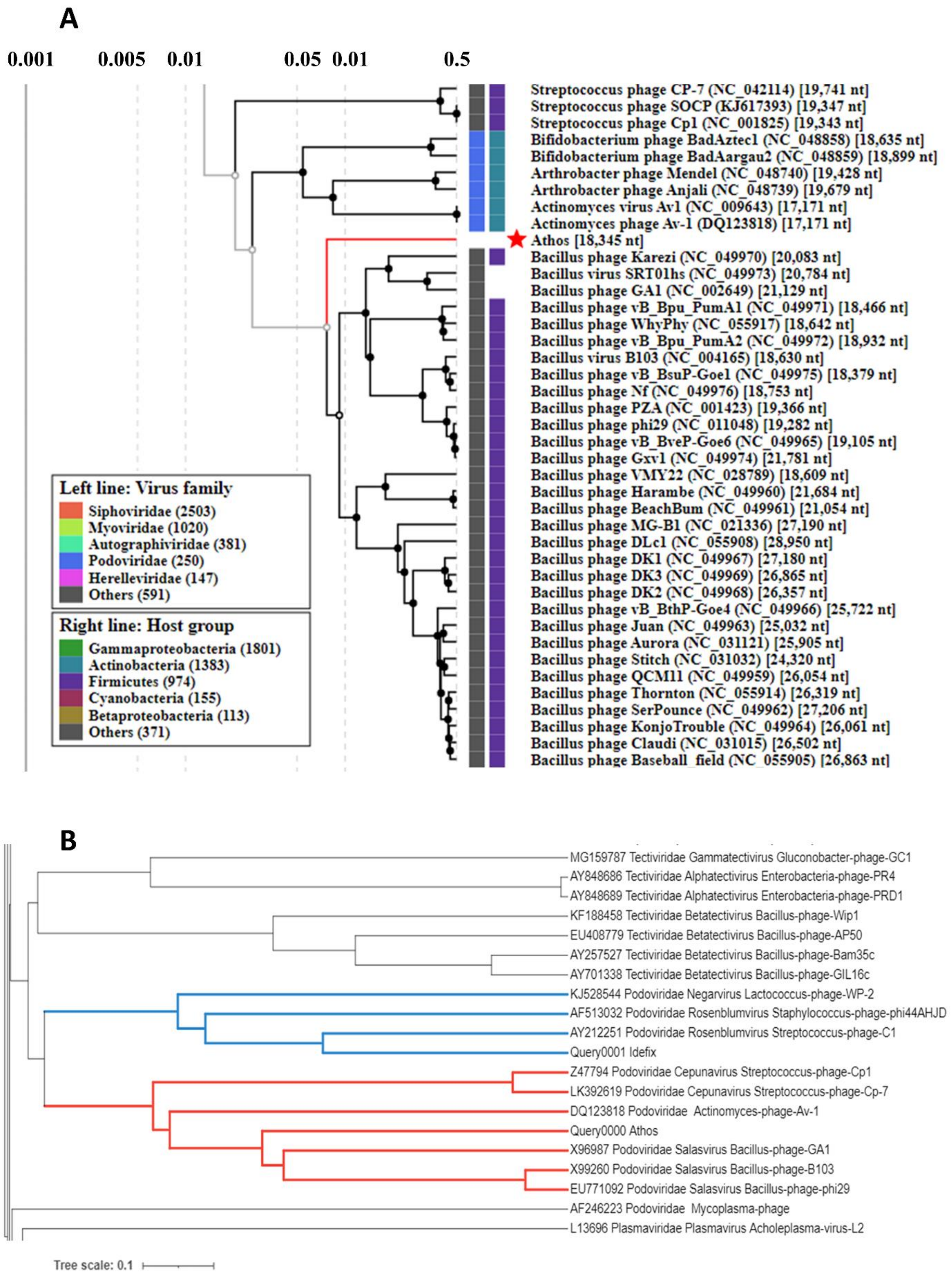

Figure S4. Placement of *Enterococcus* phage Athos in dendrograms generated with VIPTree (A) and GRAViTy (B). In the dendrogram A, generated by whole proteome tBLASTx similarity (49), only the relevant region of the tree corresponding to the *Salasmaviridae* is shown and Athos is labeled with a red star. In the dendrogram B, which is based on hidden Markov and genomic organization models (50, 51), a focus is made on the separation between the families *Rountreeviridae* in blue and *Salasmaviridae*, which includes Athos, in red.

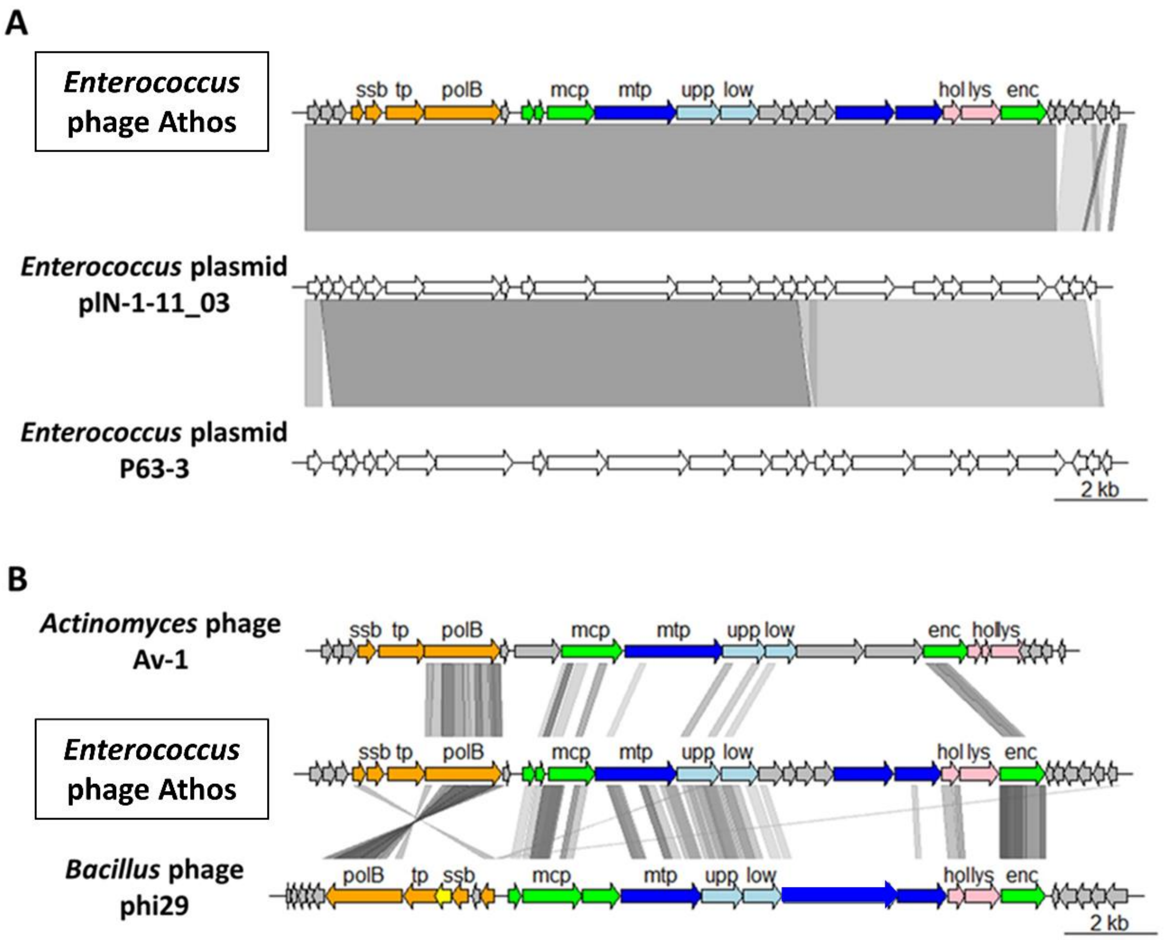

Figure S5. Annotation and comparison of *Enterococcus* phage Athos genome with "plasmids" pIN-1-11_03 and p63-3 (A) or with *Actinomyces* phage Av-1 and *Bacillus* phage phi29 (B). Genkank accession numbers of these mobile genetic elements are listed in Table S3. Genome comparisons were performed using genoplotR (46). Shed of gray lines connect regions of adjacent mobile genetic elements that have respectively BLASTn (A) or tBLASTx (B) similarity from 30% to 100% (over 100 bp for (B)). Gene functions are color-coded: orange, DNA metabolism; green, DNA packaging and head; dark blue, tail; light blue, connector; pink, lysis; yellow, transcriptional regulation; gray, hypothetical proteins; white, unannotated in A. Selected Athos genes are annotated and abbreviated: ssb, single-strand DNA-binding protein; tp, terminal protein; polB, B family polymerase; mcp, major capsid protein; mtp, major tail protein; upp, upper collar protein; low, lower collar protein; hol, holin; lys, endolysin; enc, encapsidation protein.

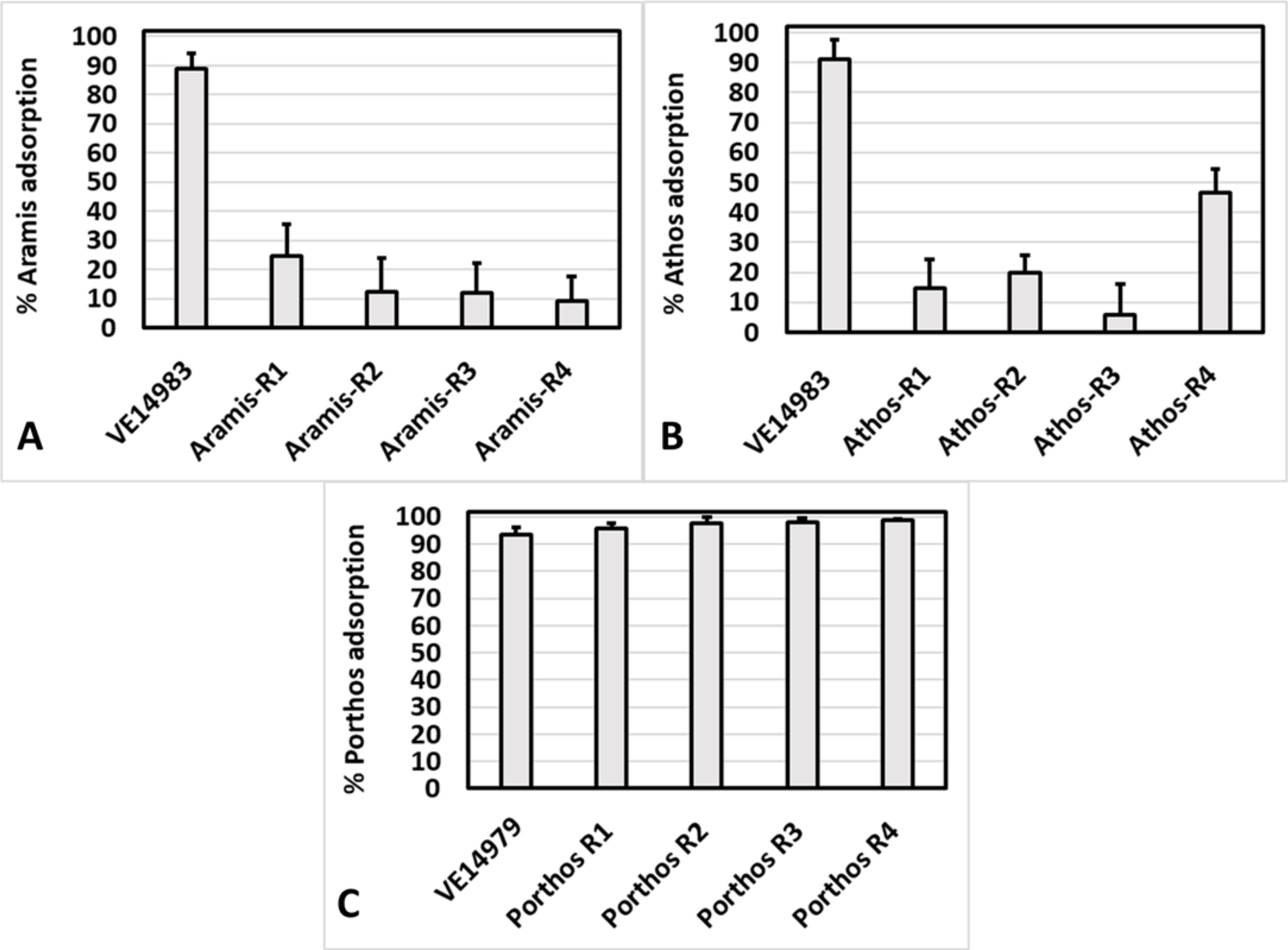

Figure S6: Adsorption of Aramis (A), Athos (B) and Porthos (C) phages to their isolation strains and their respective resistant mutants. These experiments were independently conducted at least three times and average values are given with standard deviations.

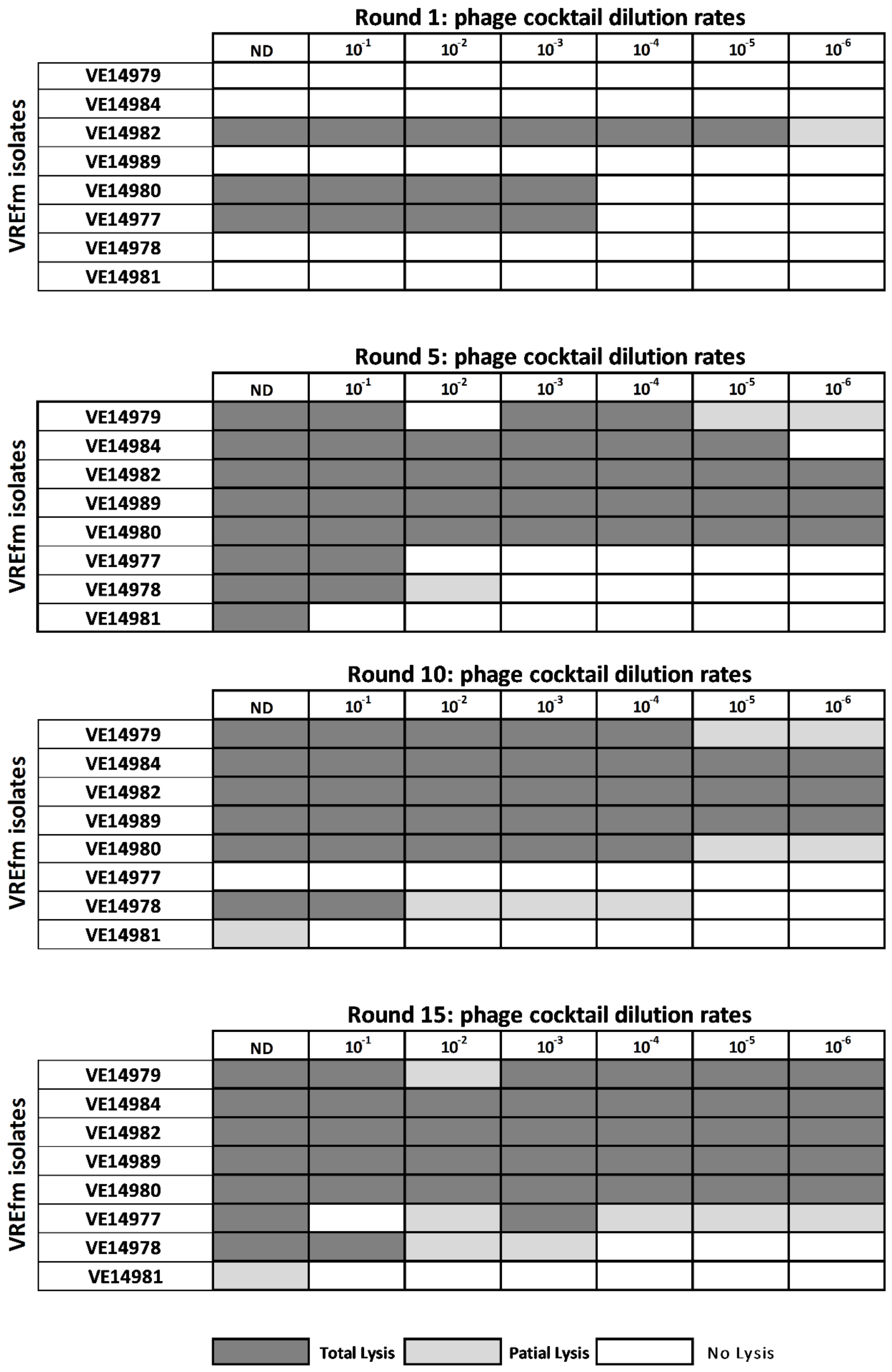

Figure S7: Progression of the phage cocktail on the eight naïve development strains every five passages.

K35.2

evidence	position	mutation	annotation	gene	encoded protein
RA	11,441	T→C	intergenic (-36/+40)	*PORT_31* ← / ← *PORT_32*	Hypothetical protein/Hypothetical protein
RA	35,388	G→A	intergenic (-16/+293)	*PORT_69* ← / ← *unknown*	Hypothetical protein/tRNA-Gly-TCC
RA	49,306	T→C	D586D (GAT→GAC)	*PORT_82* →	Terminase, large subunit
RA	81,045	G→A	G2262D (GGT→GAT)	*PORT_106* →	Tail fiber protein
RA	83,726	G→A	G637D (GGT→GAT)	*PORT_107* →	Tail protein, putative tail fiber protein
RA	83,734	G→A	G640R (GGA→AGA)	*PORT_107* →	Tail protein, putative tail fiber protein
RA	84,930	G→A	D236N (GAT→AAT)	*PORT_108* →	Tail fiber protein
RA	106,817	C→T	A84V (GCT→GTT)	*PORT_124* →	Putative antisigma factor
RA	121,753	G→T	E13* (GAA→TAA)	*PORT_145* →	Virion structural protein with Ig domain

K36.1

evidence	position	mutation	annotation	gene	encoded protein
RA	11,441	T→C	intergenic (-36/+40)	*PORT_31* ← / ← *PORT_32*	Hypothetical protein/Hypothetical protein
RA	49,306	T→C	D586D (GAT→GAC)	*PORT_82* →	Terminase, large subunit
RA	80,738	G→A	D2160N (GAC→AAC)	*PORT_106* →	Tail fiber protein
RA	83,600	G→A	R595H (CGT→CAT)	*PORT_107* →	Tail protein, putative tail fiber protein
RA	83,726	G→A	G637D (GGT→GAT)	*PORT_107* →	Tail protein, putative tail fiber protein
RA	106,817	C→T	A84V (GCT→GTT)	*PORT_124* →	Putative antisigma factor
RA	121,753	G→T	E13* (GAA→TAA)	*PORT_145* →	Virion structural protein with Ig domain

K36.3

evidence	position	mutation	annotation	gene	encoded protein
RA	35,388	G→A	intergenic (-16/+293)	*PORT_69* ← / ← *unknown*	Hypothetical protein/tRNA-Gly-TCC
RA	46,070	G→A	H22Y (CAT→TAT)	*PORT_77* ←	Hypothetical protein
RA	80,738	G→A	D2160N (GAC→AAC)	*PORT_106* →	Tail fiber protein
RA	83,600	G→A	R595H (CGT→CAT)	*PORT_107* →	Tail protein, putative tail fiber protein
RA	83,726	G→A	G637D (GGT→GAT)	*PORT_107* →	Tail protein, putative tail fiber protein
RA	106,817	C→T	A84V (GCT→GTT)	*PORT_124* →	Putative antisigma factor
RA	121,753	G→T	E13* (GAA→TAA)	*PORT_145* →	Virion structural protein with Ig domain

K41.1

evidence	position	mutation	annotation	gene	encoded protein
RA	34,164	C→A	E85* (GAA→TAA)	*PORT_68* ←	RNA binding protein
RA	34,564	$(G)_{11\rightarrow 10}$	intergenic (-148/+584)	*PORT_68* ← / ← *PORT_69*	RNA binding protein/Hypothetical protein
RA	80,738	G→A	D2160N (GAC→AAC)	*PORT_106* →	Tail fiber protein
RA	83,726	G→A	G637D (GGT→GAT)	*PORT_107* →	Tail protein, putative tail fiber protein
RA	83,734	G→A	G640R (GGA→AGA)	*PORT_107* →	Tail protein, putative tail fiber protein
RA	84,930	G→A	D236N (GAT→AAT)	*PORT_108* →	Tail fiber protein
RA	106,817	C→T	A84V (GCT→GTT)	*PORT_124* →	Putative antisigma factor
RA	118,113	C→T	S155L (TCA→TTA)	*PORT_140* →	Single-stranded DNA binding protein

Figure S8. Base substitution mutations and small indels predicted in K35.2, K36.1, K36.3 and K41.1. These predictions were made by examining the pileup of the respective evolved phage cleaned reads mapped to each position in the Porthos reference genome using the consensus mode of breseq tool (52). Each mutation/indel were predicted thanks to read alignment evidence (evidence column, RA) and listed with their position in comparison with the reference genome (position column); their nature (mutation column); their consequence (annotation column, nonsense, silent and missense substitutions are respectively detailed in red, green and blue); the gene and protein concerned or the intergenic region (gene and encoded protein columns).

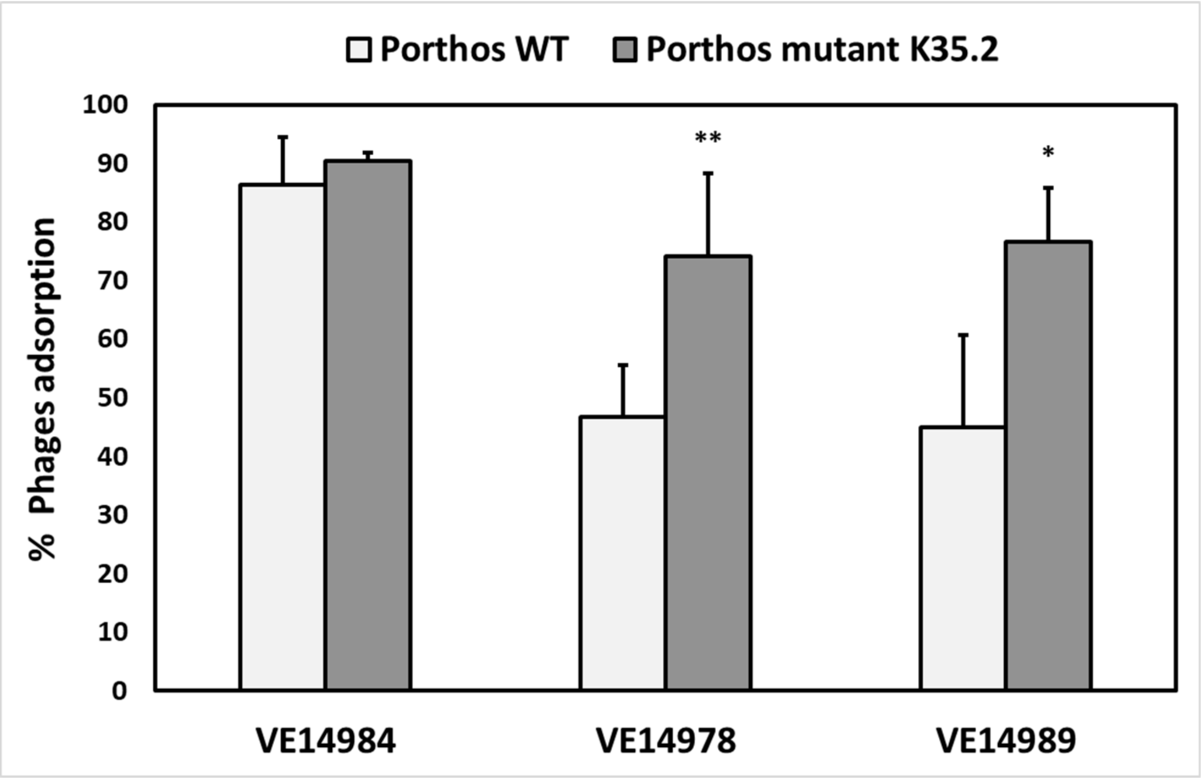

Figure S9: Adsorption of Porthos phage and its derived mutant K35.2 to the strains VE14984 (on which both phages grew as efficiently), VE14978 and VE14989 (both only sensitive to K35.2). These experiments were independently conducted four times and average values are given with standard deviations. *, P<0.05; **, P<0.01; by unilateral unpaired Student's t test.

Supplemental tables

Table S1. Description of VREfm clinical isolates used to constitute the bacteriophage collection and evaluate phage host spectra.

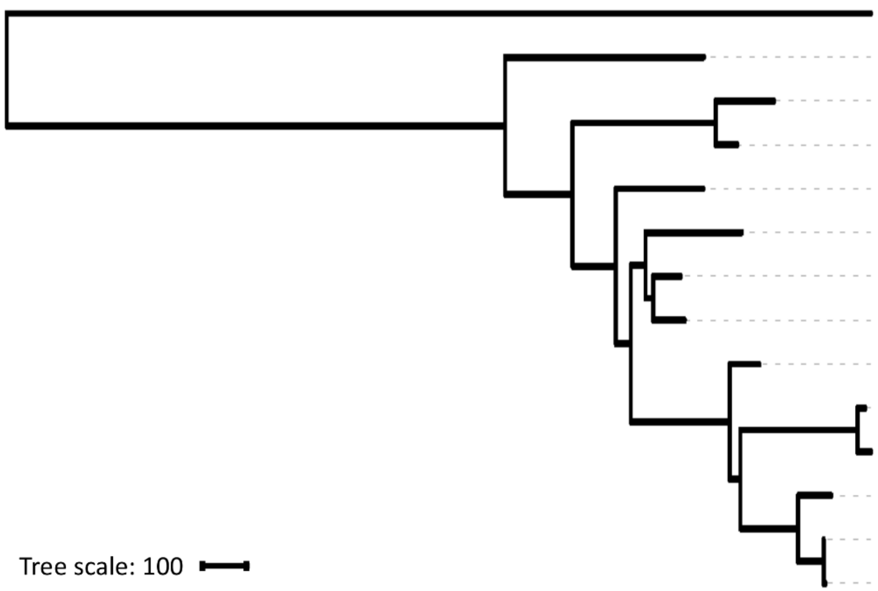

Micalis code	CNR name	City	Country	Year	ST	*van* gene
VE14976	06-007	Clermont-Ferrand	France	2006	ST25	*vanA*
VE14980	06-084	Brest	France	2006	ST18	*vanA*
VE14979	06-055	Sarrebourg	France	2006	ST280	*vanA*
VE14988	09-122	Vichy	France	2009	ST280	*vanA*
VE14978	06-047	Colmar	France	2006	ST78	*vanA*
VE14977	06-046	Paris	France	2006	New	*vanA*
VE14983	07-095	Clichy	France	2007	ST17	*vanA*
VE14989	Aus0004	Melbourne	Australia	1998	ST17	*vanB*
VE14985	09-001	Caen	France	2009	ST17	*vanB*
VE14981	07-018	Toulouse	France	2007	ST203	*vanB*
VE14987	09-087	Paris	France	2009	ST2052	*vanA*
VE14982	07-079	Strasbourg	France	2007	ST202	*vanB*
VE14984	08-225	Béthune	France	2008	ST192	*vanB*
VE14986	09-038	Barlin	France	2009	ST192	*vanB*

The phylogenetic neighbor-joining tree based on the core genome SNPs was generated with iTOL (31). CNR, Centre National de Référence; ST, Sequence Type; *van* gene, vancomycin-resistance operon.

Table S2. Description of *E. mundtii*, *E. durans*, *E. hirae* and *E. faecalis* strains used to evaluate the host spectra of the five natural phages characterized in details, as well as those of two of the five evolved phages emerged from the Appelmans experiment.

Micalis Code	Reference or Source	Other code	Isolation year	Origin	Isolation country	ST	Species
VE14732	(90)	LMG10748T	<1971	Soil	USA		*E. mundtii*
VE14719	CHV (Versailles, France)	CHV7805	<2004	Clinical	France		*E. durans*
VE14730	(91)	LMG10746T	<1937	Dried Milk	Unknown		
VE14795	INRA (Corte, France)	CE30	1982	Goat cheese	France		
VE14797	INRA (Corte, France)	CE183	1982	Presure	France		
VE19203			2020	Poultry	France		
VE14708	CHV (Versailles, France)	CHV6605	<2004	Clinical	France		*E. hirae*
VE14731		LMG6399T	<1949	Unknown	Unknown		
VE14740			1989	Food	France		
VE14767		23D1	2004	Mouse gut	France		
VE14806	INRA (Corte, France)	CE130	1982	Food	France		
VE14945			2014	Mouse gut	France		
VE14794	INRA (Corte, France)	CE22	1982	Food	France	279	*E. faecalis*
VE14000	(92)	JH2-2	1973	Clinical	UK	8	
VE14001	(93)	OG1RF	1978	Clinical	USA	1	
VE14633	INRA (Aurillac, France)	1085	1991	Food	France	277	
VE14568	Health Sciences Center (Oklahoma City, USA)	609	<1992	Clinical	USA	19	
VE14820	AP-HP, Hôpital Européen Georges Pompidou, Paris, France		2005	Clinical	France	281	
VE14840	(94)	F11/KA1.2	2005	Food	France	21	
VE14578	Health Sciences Center (Oklahoma City, USA)	647	1994	Commensal	USA	275	
VE14002	(95)			Clinical	USA	6	

ST, Sequence Type.

90. Collins MD, Farrow JAE, Jones D. 1986. *Enterococcus mundtii* sp. nov. Int J Syst Bacteriol 36:8–12.
91. Collins MD, Jones D, Farrow JAE, Kilpper-Balz R, Schleifer KH. 1984. *Enterococcus avium* nom. rev., comb. nov.; *E. casseliflavus* nom. rev., comb. nov.; E. *durans* nom. rev., comb. nov.; *E. gallinarum* comb. nov.; and *E. malodoratus* sp. nov. Int J Syst Bacteriol 34:220–223.
92. Jacob AE, Hobbs SJ. 1974. Conjugal Transfer of Plasmid-Borne Multiple Antibiotic Resistance in *Streptococcus faecalis* var. *zymogenes*. J Bacteriol 117:360–372.
93. Dunny GM, Brown BL, Clewell DB. 1978. Induced cell aggregation and mating in *Streptococcus faecalis*: evidence for a bacterial sex pheromone. Proc Natl Acad Sci USA 75:3479–3483.
94. Jamet E, Akary E, Poisson M-A, Chamba J-F, Bertrand X, Serror P. 2012. Prevalence and characterization of antibiotic resistant *Enterococcus faecalis* in French cheeses. Food Microbiol 31:191–198.
95. Sahm DF, Kissinger J, Gilmore MS, Murray PR, Mulder R, Solliday J, Clarke B. 1989. In vitro susceptibility studies of vancomycin-resistant *Enterococcus faecalis*. Antimicrob Agents Chemother 33:1588–1591.

Table S3. Genbank accession numbers of all phage genomes and plasmids used in this study for comparative genomics and/or taxonomic classification

Phage genome	Accession number
Enterococcus phage Porthos	LR990835
Enterococcus phage dArtagnan	LR991625
Enterococcus phage Aramis	LR990833
Enterococcus phage Athos	LR990834
Enterococcus phage Planchet	OX422066
Enterococcus phage vB_OCPT_CCS1	ON113170
Enterococcus phage vB_OCPT_Ben	MN027503
Enterococcus phage vB_OCPT_Bop	ON125307
Enterococcus phage vB_OCPT_Bill	OM966901
Enterococcus phage phiSHEF16	OL799260
Enterococcus phage phiSHEF13	OL799258
Enterococcus phage 113	MZ147816
Enterococcus phage If6	MT909815
Enterococcus phage EFP01	NC_047796
Enterococcus phage PEf771	MN241318
Enterococcus phage EFGrNG	MW004545
Enterococcus phage EFGrKN	MW004544
Enterococcus phage GVEsP-1	MZ333462
Enterococcus phage EfV12-phi1	NC_048087
Enterococcus phage UTI-EfS3	OL870611
Enterococcus phage vB_EfaM_A2	MT856905
Enterococcus phage EFDG1	NC_029009
Enterococcus phage IME-EFm1	KJ010489
Enterococcus phage IME-EFm5	KT588072
Enterococcus phage vB_Efm_LG62	OP018674
Enterococcus phage vB_EfaS-DELF1	LC513943
Enterococcus phage 9184	MT939242
Enterococcus phage 9183	MT939241
pIN-1-11_03	CP063571
p63-3	CP019991
Streptococcus phage C1	AY212251
Enterococcus phage Idefix	LT630001
Staphylococcus phage phiP68	AF513033
Streptococcus phage Cp-1	NC_001825
Bacillus phage phi29	NC_011048
Actinomyces phage Av-1	DQ123818

Table S4. Details about the isolation of the 21 phages isolated using VREfm isolates.

***E. faecium* strain**	**Phage source**	**Plaque morphology**	**Phage number**
VE14979 (ST280)	Wastewater sample B2	Clear ; Ø < 1mm	2
	Wastewater sample B5	Clear ; Ø < 1 mm	4 (Porthos)
	Wastewater sample V	Turbid ; Ø < 1 mm	5
VE14982 (ST202)	Wastewater sample B2	Clear ; Ø 1-2 mm	6 (dArtagnan)
	Wastewater sample B3	Bull's eye morphology ; Ø < 1mm	7
	Wastewater sample V	Turbid ; Ø < 1mm	9
VE14983 (ST17)	Wastewater sample B2	Bull's eye morphology ; Ø 1 mm	16 (Porthos)
	Wastewater sample B2	Clear ; Ø 1-2 mm	26
	Wastewater sample B3	Clear ; Ø 1-2 mm	21
	Wastewater sample B3	Bull's eye morphology ; Ø < 1-1 mm	17
	Wastewater sample B5	Clear ; Ø 1-2 mm	18 (Aramis)
	Wastewater sample V	Clear ; Ø 1-2 mm	19
VE14984 (ST192)	Wastewater sample B3	Turbid ; Ø < 1 mm	23
VE14986 (ST192)	Wastewater sample B2	Turbid ; Ø < 1 mm	22
VE14989 (ST17)	Wastewater sample B2	Turbid ; Ø 1-2 mm	24
	Wastewater sample B2	Clear ; Ø 1-2 mm	10
	Wastewater sample B2	Clear ; Ø 2-3 mm	20
	Wastewater sample B3	Clear ; Ø 3 mm	12
	Wastewater sample B3	Clear ; Ø 2-3 mm	13
	Wastewater sample B5	Clear Ø 2-3 mm	11
VE14980 (ST18)	Pig fecal virome sample VP7	Clear Ø 1 mm	27

ST, sequence type; Ø, diameter; bull's eye morphology, plaque center clearer than the outer edges.

Table S5. Host ranges of the collection of VREfm phages.

Isolated phages	VREfm isolates (Sequence Type)													
	VE14979 (ST280)	VE14982 (ST202)	VE14983 (ST17)	VE14984 (ST192)	VE14985 (ST17)	VE14986 (ST192)	VE14988 (ST280)	VE14989 (ST17)	VE14980 (ST18)	VE14977 (New ST)	VE14976 (ST25)	VE14978 (ST78)	VE14981 (ST203)	VE14987 (ST2052)
16 (Athos)		[light gray]	[dark gray] *		[light gray]									
4 (Porthos)	[dark gray] *		[light gray]	[dark gray]		[dark gray]	[dark gray]							
2	[dark gray] *			[light gray]		[light gray]								
18 (Aramis)		[light gray]	[dark gray] *					[dark gray]						
19			[dark gray] *					[light gray]						
21		[light gray]	[dark gray] *					[light gray]						
24		[light gray]	[light gray]					[dark gray] *						
26		[light gray]	[dark gray] *					[light gray]						
6 (dArtagnan)		[dark gray] *		[light gray]	[light gray]			[dark gray]						
11			[dark gray]					[dark gray] *						
10		[light gray]						[dark gray] *						
13		[light gray]						[dark gray] *						
7		[dark gray] *			[light gray]									
9		[light gray] *			[light gray]									
22				[light gray]		[light gray] *								
23				[light gray] *		[light gray]								
12								[dark gray] *						
20								[dark gray] *						
17			[dark gray] *											
5	[dark gray] *													
27 (Planchet)			[dark gray]		[light gray]				[dark gray] *	[light gray]				

Host ranges were evaluated by spot assays on the 14 VREfm isolates. Dark gray, strong lytic activity; light gray, weak lytic activity; white, no apparent productive lytic activity. Respective isolation strains are indicated by an asterisk.

Table S6. Morphological characterization of the phages in transmission electron microscopy.

Phage number	Morphological classification	Average head diameter (sd)	Average head length (sd)	Average tail diameter (sd)	Average tail length (sd)
6	siphophage	63.1 nm (1.8)	63.6 nm (3.1)	10.4 nm (1.2)	230.3 nm (5.0)
12	siphophage	53.9 nm (4.2)	53.1 nm (2.7)	10.5 nm (1.0)	210.1 nm (11.4)
18	siphophage	57.2 nm (2.3)	56.2 nm (2.8)	10.01 nm (1.6)	241.0 nm (4.5)
11	siphophage	59.0 nm (2.9)	60.2 nm (2.6)	11.4 nm (1.4)	247.8 nm (8.8)
13	siphophage	53.6 nm (4.1)	55.8 nm (3.4)	10.0 nm (1.5)	231.0 nm (8.6)
27	siphophage	69.6 nm (2.0)	71.4nm (4.2)	10.6 nm (0.9)	175.8 nm (13.2)
23	siphophage	89.0 nm (4.3)	85.5 nm (2.3)	13.7 nm (0.5)	455.4 nm (7.1)
2	myophage	99.1 nm (12.1)	99.7 nm (12.6)	21.3 nm (2.5)	232.9 nm (10.0)
4	myophage	99.1 nm (1.9)	100.6 nm (4.3)	24.1 nm (2.9)	230.0 nm (9.0)
16	podophage	42.0 nm (3.1)	64.3 nm (2.4)	11.1 nm (2.4)	29.9 nm (4.5)
7	podophage	46.4 nm (2.1)	63.9 nm (3.4)	12.5 nm (3.0)	33.9 nm (4.2)

Measurements were performed using ImageJ (37) on five virions for each phage. Average values are given with standard deviations (sd).

Table S7. Host ranges of Porthos, dArtagnan, Aramis, Planchet and Athos on a set of 21 tested enterococcal isolates other than *E. faecium*.

			Phages				
			**Porthos**	**dArtagnan**	**Aramis**	**Planchet**	**Athos**
Enterococcus	***E. durans***	**VE14719**					
		**VE14730**					
		**VE14795**					
		**VE14797**					
		**VE19203**					
	E. hirae	**VE14708**					
		**VE14767**					
		**VE14945**					
		**VE14740**					
		**VE14806**					
		**VE14731**					
	E. mundtii	**VE14732**					
	E. faecalis	**VE14000**					
		**VE14001**					
		**VE14568**					
		**VE14820**					
		**VE14002**					
		**VE14578**					
		**VE14794**					
		**VE14633**					
		**VE14840**					

Host ranges were evaluated by spot assays on five *E. durans*, six *E. hirae*, one *E. mundtii* and nine *E. faecalis* isolates. Dark gray, strong lytic activity; light gray, weak lytic activity; white, no apparent productive lytic activity.

Table S8. Theoretical spectrum of activity of the 5 phages in mixture against the panel of 14 VREfm tested, based on individual phage plaquing host ranges.

	Phages				
VREfm isolates	dArtagnan	Porthos	Planchet	Aramis	Athos
VE14982					
VE14985					
VE14989					
VE14979					
VE14984					
VE14988					
VE14986					
VE14983					
VE14977					
VE14980					
VE14978					
VE14976					
VE14981					
VE14987					

Host ranges were evaluated by spot assays. Dark gray, strong lytic activity; light gray, weak lytic activity; white, no apparent productive lytic activity.

Table S9. Resistance and crossed resistance evaluation of the spontaneous phage-resistant mutants to Aramis, Athos, Porthos and Planchet by spot assays.

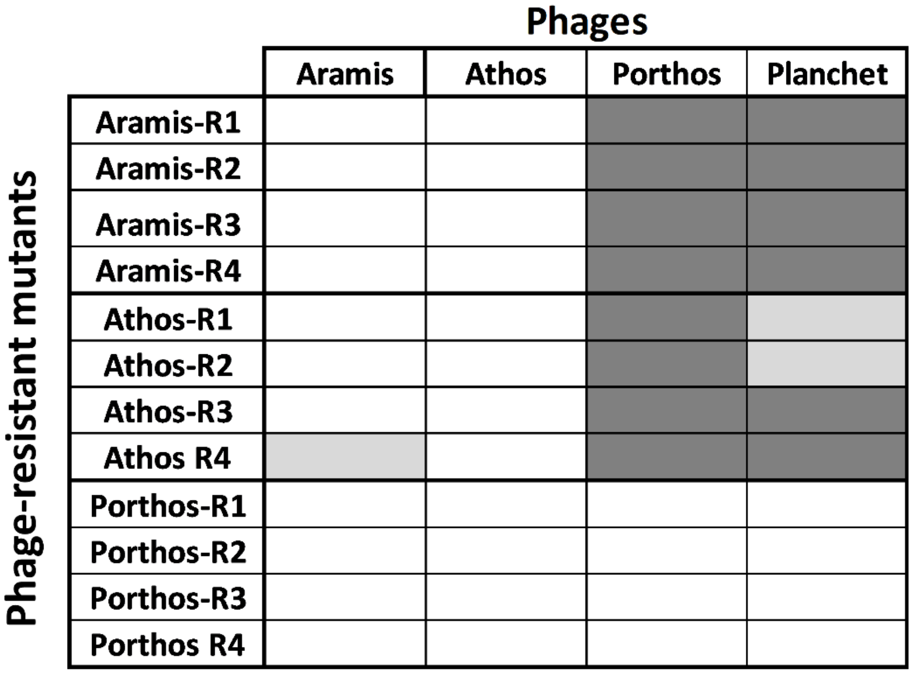

Dark gray, same amount (efficiency of plating ~10-100%) and same morphology of plaques obtained on mutants and the corresponding wild-type strain, no (significant) phage-resistance of the mutants; light gray, turbid plaques (efficiency of plating ~1%) on the spontaneous mutant compared the clear plaques obtained on the wild-type strain, weak phage-resistance of the mutant; white, no apparent productive lytic activity, strong phage-resistance of the mutants.

Table S10. Point mutations found with breseq (52) in the variable part of *epa* locus in Aramis- and Athos-respective primary adsorption resistant mutants, which were always susceptible to Porthos.

**Spontaneous phage-resistant mutant**	**CDS Function Position**	**Mutation type**	**Reference**	**Mutation**	**Amino acids change**	**Resistance to Aramis Athos Porthos**
Aramis-R1	Glycosyltransferase 26510	SNP	C	G	Missense mutation His/Asp	+ + -
Aramis-R2	O-antigen ligase 28641	Del	T		Frameshift Premature stop codon	+ + -
Aramis-R3	O-antigen ligase 28626	Del	T		Frameshift Premature stop codon	+ + -
Aramis-R4	O-antigen ligase 28586	SNP	G	T	Nonsense mutation	+ + -
Athos-R1	Glycosyltransferase 24330	Del	A		Frameshift Premature stop codon	+ + -
Athos-R2	Hexose epimerase 40220	SNP	G	T	Nonsense mutation	+ + -
Athos-R3	O-antigen ligase 28978	Del	A		Frameshift Premature stop codon	+ + -
Athos-R4	O-antigen ligase 28601	Del	A		Nonsense mutation	+/- + -

CDS, coding sequence; SNP, single nucleotide polymorphism; Del, deletion; +, strong phage-resistance of the mutants; +/-, weak phage-resistance of the mutants; -, no (significant) phage-resistance of the mutants.

Table S11. Antibiotic susceptibility of phage-resistant *E. faecium* mutants compared to their corresponding parental strains.

Strain	Inhibition diameter (mm)[a]											MIC (mg/L)[b]									
	API	IPM	GME	CHL	ERY	CMN	DQU	RIF	TET	NOR	SXT	VAN	TEC	DAP	TGC	LZD	TDZ	TEL	DAL	ORI
VE14983 WT	6	6	20	25	6	6	28	15	8	6	29	128	64	4	0.12	1	0.25	1	0.5	0.01
Athos-R1	6	6	23	26	6	6	31	17	8	6	30	128	**16**	**1**	0.06	1	0.25	1	1	0.01
Athos-R2	6	6	22	25	6	6	30	16	8	6	30	64	**16**	**1**	0.06	1	0.25	2	2	0.01
Athos-R3	6	6	20	25	6	6	29	16	7	6	30	64	**4**	**0.5**	0.06	1	0.25	**0.25**	1	0.01
Athos-R4	6	6	22	25	6	6	28	14	8	6	28	64	**16**	4	0.12	1	0.25	0.5	0.25	<0.01
Aramis-R1	6	6	23	27	6	6	30	15	9	6	28	128	**16**	**1**	0.06	1	0.25	0.5	0.25	<0.01
Aramis-R2	6	6	22	30	6	6	32	17	9	6	31	128	**8**	4	0.12	1	0.25	**0.25**	**0.12**	<0.01
Aramis-R3	6	6	23	28	6	6	30	17	8	6	29	64	**8**	4	0.06	1	0.25	1	0.25	<0.01
Aramis-R4	6	6	21	28	6	6	30	16	8	6	29	64	**8**	4	0.06	1	0.25	0.5	0.5	<0.01
VE14979 WT	6	6	8	26	6	6	28	6	7	6	23	>256	32	2	0.25	2	0.5	4	8	0.06
Porthos-R2	6	6	6	28	6	6	30	6	8	6	25	>256	16	2	0.12	2	0.25	4	4	0.03

[a]Antibiotics tested by disk diffusion: API, ampicillin; CHL, chloramphenicol; CMN, clindamycin; DQU, dalfopristin-quinupristin; ERY, erythromycin; GME, gentamicin; NOR, norfloxacin; IPM, imipenem; RIF, rifampicin; SXT, cotrimoxazole; TET, tetracycline.

[b]Antibiotics tested by MIC determination: DAL, dalbavancin; DAP, daptomycin; LZD, linezolid; ORI, oritavancin; TDZ, tedizolid; TEC, teicoplanin; TEL, telavancin; TGC, tigecycline; VAN, vancomycin. Significant changes in MICs (≥4 fold) are indicated in bold.

Table S12 Emergence of VE14983 spontaneous CFUs following teicoplanin, Aramis and teicoplanin/Aramis exposures.

**Exposure**	Teicoplanin (64 µg/mL)	Aramis phage (MOI ~ 50)	Teicoplanin (64 µg/mL) Aramis phage (MOI ~ 50)
**Frequency of emergence of spontaneous CFUs (sd)**	1.25 (± 0.6) x 10^{-5}	2.6 (± 1.9) x 10^{-5}	0

The frequency of emergence of VE14983 spontaneous CFUs was estimated following three independent experiments and average values are given with standard deviations (sd).

Table S13. Isolated and purified phages from the evolved cocktail per development strain.

Development strain	Number of isolated and purified phages from the evolved cocktail
VE14979	2
VE14984	3
VE14982	3
VE14989	1
VE14980	3
VE14977	0
VE14978	3
VE14981	3

Table S14. Comparison between the host ranges of the wild-type phages present in the cocktail at the starting point and those of the 18 phages purified from the cocktail at the ending point.

	**Wild type phage in the starting cocktail**				**Purified phages from the evolved cocktail**																	
Isolate	Porthos	dArtagnan	Aramis	Planchet	K.35.1	**K.35.2**	K.35.3	**K.36.1**	**K.36.3**	**K.41.1**	K.41.2	K.41.3	K.39.1	K.39.2	K.39.3	K.46.1	K.37.1	K.37.2	K.37.3	K.38.1	**K.38.2**	K.38.3
VE14979																						
VE14984																						
VE14982																						
VE1989																						
VE14980																						
VE14977																						
VE14978																						
VE14981																						
**Total**	2	3	1	2	3	**5**	2	**5**	**5**	**5**	3	2	3	3	3	3	1	2	2	2	**2**	2

Host ranges were evaluated by spot assays on the eight development strains used during the evolution experiment. Dark gray, strong lytic activity; light gray, weak lytic activity; white, no apparent productive lytic activity.

Table S15. Comparison between the host ranges of the wild-type phages present in the cocktail at the starting point and those of the five selected evolved phages purified from the cocktail at the ending point.

		Wild type phages				Evolved phages				
		Porthos	dArtagnan	Aramis	Planchet	K35.2	K36.1	K36.3	K41.1	K38.2
Development strains	VE14979									
	VE14984									
	VE14982									
	VE14989									
	VE14980									
	VE14977									
	VE14978									
	VE14981									
Remaining strains	VE14988									
	VE14986									
	VE14983									
	VE14985									
	VE14976									
	VE14987									
	Total	5	4	2	4	9	9	9	10	3

Host ranges were evaluated by spot assays on both the eight development strains and the remaining VREfm strains from our collection that were not used for the evolution experiment. Dark gray, strong lytic activity; light gray, weak lytic activity; white, no apparent productive lytic activity.

Table S16. Comparison between the host ranges of Porthos and its derivates K35.2 and K41.1 on enterococci species distinct from *E. faecium*.

			**WT**	**Mutants**	
			**Porthos**	**K35.2**	**K41.1**
Enterococcus	***E. durans***	**VE14719**			
		**VE14730**			
		**VE14795**			
		**VE14797**			
		**VE19203**			
	E. hirae	**VE14708**			
		**VE14767**			
		**VE14945**			
		**VE14740**			
		**VE14806**			
		**VE14731**			
	E. mundtii	**VE14732**			
	E. faecalis	**VE14000**			
		**VE14001**			
		**VE14568**			
		**VE14820**			
		**VE14089**			
		**VE14578**			
		**VE14794**			
		**VE14633**			
		**VE14840**			

Host ranges were evaluated by spot assays on five *E. durans*, six *E. hirae*, one *E. mundtii* and nine *E. faecalis* isolates. Dark gray, strong lytic activity; light gray, weak lytic activity; white, no apparent productive lytic activity.
